# Investigating Short-Chain Chlorinated Paraffins (SCCPs) in China: A Review of Occurrences, Determination Techniques, Human Exposure Routes, Toxicity, and Risk Assessments

**DOI:** 10.3390/toxics14070567

**Published:** 2026-06-27

**Authors:** Jiangbo Niu, Zixuan Qiu, Jiaying Yang, Shuren Liu, Lili Niu, Zili Guo, Shuang Zhang, Shuduan Mao, Weiping Liu

**Affiliations:** 1 College of Environment, Zhejiang University of Technology, Hangzhou 310032, China; 18703734809@163.com; 2 Zhejiang Collaborative Innovation Center for Full-Process Monitoring and Green Governance of Emerging Contaminants, Interdisciplinary Research Academy (IRA), Zhejiang Shuren University, Hangzhou 310015, China; qzx20050714@163.com (Z.Q.); jiannie3412@163.com (J.Y.); liushuren@zjsru.edu.cn (S.L.); niulili@zjsru.edu.cn (L.N.); guozili@zjsru.edu.cn (Z.G.); zhangshuang@zjsru.edu.cn (S.Z.); wliu@zju.edu.cn (W.L.)

**Keywords:** short-chain chlorinated paraffins, environmental distribution, human exposure, toxicity, adverse health effects

## Abstract

Chlorinated paraffins (CPs) are recognized as a novel class of persistent organic pollutants (POPs) and are categorized into short- (SCCPs, C_10–13_), medium- (MCCPs, C1_4–17_), and long- (LCCPs, C_≥18_) chain CPs considering the carbon-chain length. Among them, SCCPs possess lower molecular weights, higher vapor pressures, and greater water solubilities compared to their longer-chain counterparts (MCCPs and LCCPs), which promote their environmental release. Consequently, SCCPs were designated as POPs of concern under the Stockholm Convention in 2017. This review concludes the recent research progress of SCCPs in China from 2015 to present, and we present a comprehensive overview of SCCP concentrations, encompassing diverse environmental matrices and human tissues, for example, air, water, soil, sediments, biota, food, human placenta, breast milk, blood, and organs (fat, kidney, liver, brain, bone, etc.). Whereafter, we summarize the development of SCCPs determination methods, benefiting from quantifying relative carbon-chain length and chlorine content of SCCPs correctly. Moreover, toxicity, toxicokinetics, and adverse health effects of SCCPs in humans from China are concluded and discussed. Meanwhile, we review the existing control and treatment technologies for SCCPs. Lastly, we describe some noteworthy and prospective issues that are worthy of further study. In the future, the relevant studies are still necessary to keep up with consecutive monitoring and evaluation of SCCP levels and relative potential health impacts in China.

## 1. Introduction

Chlorinated paraffins (CPs) are high-production-volume industrial chemicals comprising complex mixtures of polychlorinated n-alkanes (general formula of C_n_H_2n+2−x_Cl_x_) with a chlorine content of 30~72 wt%. CPs are classified by carbon-chain length into short-chain (SCCPs, C_10–13_), medium-chain (MCCPs, C_14–17_), and long-chain (LCCPs, C_≥18_) groups, and are extensively utilized as plasticizers and flame retardants in polymers, lubricants, sealants, and coatings [1,2]. Their inherent persistence, bioaccumulation potential, toxicity, and capacity for long-range environmental transport have led to the listing of CPs as persistent organic pollutants (POPs) under the Stockholm Convention [3]. Global production has been substantial, and cumulative releases have resulted in their widespread detection in air, water, soil, and biota [4,5,6,7,8]. Moreover, human and wildlife exposure occurs primarily via ingestion, raising serious concerns over developmental, neurological, endocrine, and metabolic toxicity [9,10,11,12,13,14,15].

Among CPs, SCCPs possess distinct properties—such as lower molecular weight, higher volatility, and greater environmental mobility—that enhance their dispersal and bioavailability [16,17,18]. Historically, produced in the largest volume among POPs, SCCPs have faced global restrictions that have accelerated the use of MCCPs and LCCPs as substitutes, despite growing recognition of their similar persistence, bioaccumulation potential, and toxicity concerns [19]. Indeed, regulatory attention is expanding, as evidenced by the European proposal to restrict MCCPs and the classification of SCCPs as a Group 2B potential carcinogen by the International Agency for Research on Cancer (IARC) [20].

While global production is shifting, China remains a pivotal region for understanding the lifecycle and impact of SCCPs. China is the world’s largest producer and consumer, with ongoing significant use despite international phase-outs [21,22]. This substantial industrial footprint, coupled with intensive applications, has resulted in severe contamination. SCCPs are detected at concentrations 2~3 orders of magnitude higher than other legacy pollutants (e.g., PAHs, PBDEs) in human blood from China, suggesting a disproportionate exposure burden [22]. In response, the Chinese government has explicitly listed SCCPs in its national “New Action Plan for New Pollutants Management” (2022), highlighting them as a priority for control [21]. Consequently, China presents a critical and urgent case study: a major emission source, a hotspot for human and ecological exposure, and a key jurisdiction enacting regulatory action. Understanding the specific distribution, pathways, health risks, and management challenges of SCCPs within China is therefore not only of regional importance but also essential for evaluating the effectiveness of global POP governance and informing mitigation strategies worldwide.

**Figure 1:**
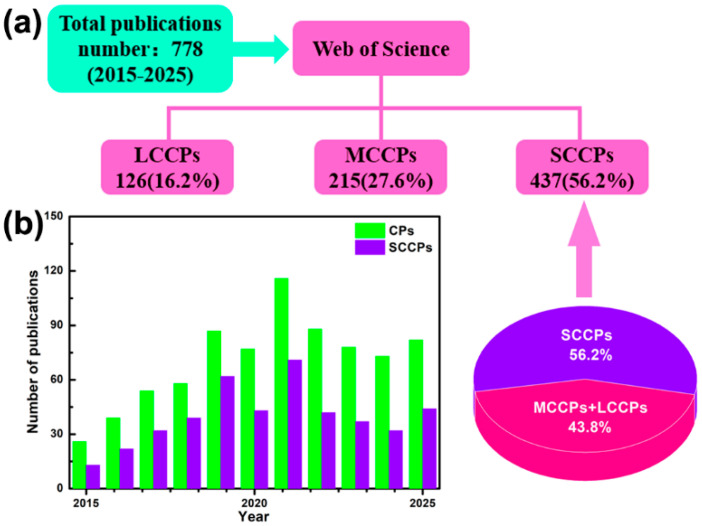
(**a**) Number of publications collected from the topics of chlorinated paraffins (CPs), LCCPs, MCCPs, and SCCPs from 2015 to 2025 (31 December 2025) using the Web of Science; (**b**) the number of published papers related to CPs and SCCPs per year from 2015 to 2025.

Although recent years have seen a remarkable increase in research on CPs (Figure 1: obtained by searching the Web of Science database using the topics “chlorinated paraffins”, “short-chain chlorinated paraffins”, “medium-chain chlorinated paraffins”, and “long-chain chlorinated paraffins” for the period 2015–2025, up to 31 December 2025), published reviews have largely focused on global perspectives, analytical methods, environmental fate, or toxicology [1,2,3,9,20,23,24,25,26,27,28,29]. For example, Chen et al. focused on biological occurrence, bioaccumulation, transmission, and metabolism of CPs [1]. Yu et al. summarized the sample treatment, instrumental characterizations, occurrence, and distribution of CPs in food samples [20]. Mu et al. reviewed the toxicity and underlying molecular mechanisms of SCCPs from a toxicological perspective [23]. Kalinowska et al. mainly focused on analytical procedures for SCCP determination [26]. Glüge et al. summarized the global production, utilization, and emission of SCCPs [28]. Despite the existence of several comprehensive reviews on CPs, a critical knowledge gap remains regarding the China-specific context, where SCCP production, usage patterns, and environmental burdens differ substantially from those in other regions. In particular, this review aims to address the following crucial China-centric scientific questions: (1) Why are SCCP concentrations in multiple environmental and human matrices in China frequently higher than those reported in Europe and North America? (2) How do China’s unique industrial structure, including e-waste recycling, plastic manufacturing, and informal sectors, cause the spatial distribution and exposure pathways of SCCPs? (3) What are the major uncertainties and regional data gaps that limit accurate risk assessment in China? (4) How effective are current Chinese regulatory actions compared to global control frameworks, and what challenges remain? To address these questions, the present review critically summarizes the literature from 2015 to 2025, with the following objectives: (1) to describe the current distribution and levels of SCCPs across key environmental matrices (air, water, soil, sediment) and human samples (blood, milk, tissues) in China; (2) to evaluate advances and challenges in analytical methods for the accurate determination of SCCPs in diverse samples; (3) to review the evidence linking SCCP exposure to potential adverse health effects; and (4) to outline the current regulatory framework and management strategies for SCCPs in China, identifying progress and remaining challenges. Consequently, this work aims to clarify the specific framework of the SCCP issue in China, identify critical research needs, and support the development of effective monitoring and regulatory interventions to protect human and environmental health.

## 2. Levels and Distribution of SCCPs in Environmental Matrices of China

This section summarizes SCCP monitoring data from Chinese environmental matrices published between 2015 and 2025. We organize the analysis by individual matrices—air (Section 2.1), water (Section 2.2), soil (Section 2.3), sediment (Section 2.4), biota (Section 2.5), and food (Section 2.6)—because SCCP distribution, transport, and accumulation differ markedly among media. For each matrix, we provide national concentration ranges and hotspots, describe spatial gradients, and, where data permit, compare Chinese levels with global benchmarks, as summarized in Table 1 (compiled from previous studies reported in Table 2, Table 3, Table 4, Table 5, Table 6 and Table 7). Previous studies have extensively summarized the environmental occurrence of SCCPs in China [25,30]. Among all media, the atmosphere is considered an extremely pivotal medium for the long-distance transport of SCCPs.

### 2.1. Air

Owing to their environmental persistence and semi-volatility, SCCPs readily enter the atmosphere at ambient temperature. They partition between gaseous and particulate phases, with monitoring data indicating a predominance in the gas phase, where concentrations exceed those in the particle phase by a factor of 0.46 to 45.7 [31,32,33,34,35,36,37]. The atmosphere is a crucial medium for the long-range environmental transport of SCCPs, and the occurrence and distribution of SCCPs in air across China are listed in Table 2. Atmospheric SCCP concentrations across China range from 0.13 to 1442 ng/m^3^, with the highest levels observed in Zibo city. Atmospheric SCCP levels exhibit distinct seasonal variations, primarily governed by temperature-driven gas–particle partitioning. Overall concentrations are typically higher in summer than in winter [36,38,39] and spring [31]. In contrast, the fraction associated with particles often shows an inverse trend [40]. This phase-specific seasonality elucidates the critical influence of temperature, with warmer conditions promoting the volatilization of SCCPs from particles into the gas phase [41]. For instance, in the Yangtze River Delta region, SCCP concentrations ranged from 6.1–63 ng/m^3^ in summer to 6.2–42 ng/m^3^ in winter [38]. A study in Jinan reported SCCP concentrations in PM_2.5_ ranging from 9.80 to 105 ng/m^3^, showing a positive correlation with PM_2.5_ levels and a negative correlation with ambient temperature, further revealing their adhesion to atmospheric particles [40].

Noticeably, spatial variability in atmospheric SCCP concentrations across China is significant and driven by regional disparities in production/consumption, physicochemical properties, and meteorological conditions [30]. In high-concentration regions, the highest levels are identified in northern and central China. Coastal areas generally present higher concentrations than central and western regions, consistent with higher GDP per capita, population density, and the geographic distribution of manufacturing plants [24]. The concentration rank is generally eastern China > northeastern China > southern China. Meanwhile, comparatively lower levels of SCCPs are found in the atmosphere of southwestern China, northwestern China, and marine areas. Background sites, such as Shergyla Mountain on the Qinghai–Tibet Plateau (130–1300 ng/m^3^), show concentrations up to one order of magnitude lower than those in emission zones like Lhasa (1100–14,440 ng/m^3^), highlighting the decisive impact of local industrial activities [42].

SCCPs are also prevalent in indoor environments, where distribution patterns are influenced by specific indoor sources such as emissions from decorative materials, furnishings, and building materials containing halogenated flame retardants [43,44,45,46]. Indoor dust acts as a reservoir for these semi-volatile compounds, leading to long-term human exposure. Concentrations indoors often exceed those of outdoors. For example, in Beijing, mean indoor SCCP levels in PM_10_, PM_2.5_, and PM_1.0_ were 61.1, 31.4, and 20.7 ng/m^3^, respectively, significantly higher than outdoor concentrations [34]. Similarly, SCCP levels on interior window films in Beijing offices (337–114,000 ng/m^3^) exceeded those on exterior films, with a gradient of offices > residential buildings > dormitories [33]. While general indoor health risks have been studied, significant occupational hazards from activities like interior finishing (e.g., cutting, sanding) remain relatively overlooked [47,48]. These processes generate abundant particulate matter that can adsorb SCCPs, posing an inhalation risk, a concern amplified by growing construction activity, which expands and prolongs workforce exposure [49,50,51]. Monitoring of complete interior finishing processes has confirmed the release and distribution of SCCPs into multiple media, including air (PM_10_, TSP) and dust [52]. Furthermore, extremely elevated SCCP levels have been reported in specific hotspots such as e-waste recycling areas and CP production plants, with concentrations markedly higher than those reported in regions like Sweden, Australia, and Canada [38,43,44,45,46,53]. Compared to reported atmospheric SCCP levels in Europe and North America, concentrations in Chinese urban and industrial regions are often one to two orders of magnitude higher. Such a discrepancy can be ascribed to the intensive production and consumption of CPs, as well as the manufacturing agglomeration in eastern China. Additionally, weaker historical emission controls and rapid urbanization have further increased atmospheric burdens.

**Table 2:** SCCP concentrations in air samples collected across various locations in China.

Region	Location	Sample	Matrices	Concentration (ng/m^3^)	Detection Method	Sampling Time	Ref.
China	10 Chinese cities ^a^	Ambient air	Particle phase (PM_2.5_)	1.98–274	HRGC-ECNI-LRMS	2013–2014	[54]
Eastern China	3 cities in the Yangtze River Delta ^b^	Ambient air	Gas phase	6.08–63.2	GC × GC-ECNI-LRMS	2011–2012	[38]
	Zibo	Inside and outside a CP production plant	Gas and particle phases	129–1442 (inside); 89.0–333 (outside)	MS-ECNI	2016	[35]
	Jinan	Ambient air	Particle phase (PM_2.5_)	9.80–105	GC-ECNI-MS	2016	[40]
	Zhoushan island	Ambient air	Gas phase	57–208	GC × GC-ECNI-MS	NA	[55]
Southern China	Shenzhen	Ambient air	Gas and particle phases	1.11–39.8	UPLC-ESI-QTOFMS	2013–2014	[5]
	Guangzhou	Indoor air	Particle phase	6.20–17.8	HRGC-ECNI-LRMS	2017	[56]
	9 cities in the Pearl River Delta ^c^	Indoor and outdoor air	Particle phase	2.90–51.8 (indoor); 1.60–32.5 (outdoor)	GC-ECNI-MS	2017	[57]
	6 cities in the Pearl River Delta ^d^	Ambient air	Particle phase (PM_2.5_)	0.832–109	UPLC-QTOF-MS	2018	[58]
	6 cities in the Pearl River Delta	Outdoor air	Submicron particulate matter (PM_1_)	8.7–89	UPLC-QTOF-MS	2018	[59]
Northern China	Beijing	Indoor air	Gas phase	60.0–1350	HRGC-ECNI-LRMS	2013–2014	[33]
	Beijing	Indoor air	Gas phase	9.77–966	GC-TOF-HRMS	2016	[46]
	Beijing	Indoor air and outdoor air	Particle phase (PM_10_)	38.3–87.7 (indoor); 16.9–28.8 (outdoor)	GC × GC-ECNI-HRTOF-MS	2016	[34]
Northeastern China	Dalian	Ambient air	Gas and particle phases	15.1–66.4 (2010); 65.3–91.0 (2016)	HRGC-ECNI-LRMS	2010 and 2016	[37]
	Dalian	Ambient air	Gas and particle phases	16.2–168	HRGC-ECNI-LRMS	2016	[32]
	Dalian	Ambient air	Gas phase	4.04–78.0	HRGC-ECNI-LRMS	2016–2017	[60]
Southwestern China	Lhasa on the Tibetan Plateau	Ambient air	Gas phase	1.10–14.4	GC-ECNI-LRMS	2012–2015	[42]
China Sea regions	Bohai Sea	Ambient air	Gas and particle phases	3.31–30.4	GC-QTOF/NCI-HRMS	2016	[31]
Background region	Shergyla Mountain on the Tibetan Plateau	Ambient air	Gas phase	0.13–1.27	GC-ECNI-LRMS	2012–2015	[42]

^a^ Beijing, Chengdu, Lanzhou, Wuhan, Taiyuan, Guiyang, Xinxiang, Guangzhou, Nanjing, and Shanghai. ^b^ Suzhou, Wuxi, and Nantong. ^c^ Guangzhou, Shenzhen, Dongguan, Foshan, Zhongshan, Zhuhai, Huizhou, Zhaoqing, and Jiangmen. ^d^ Guangzhou, Foshan, Shenzhen, Zhuhai, Zhongshan, and Maoming.

### 2.2. Water

SCCPs are characterized by low water solubility and high lipophilicity, as indicated by their logarithmic octanol–water partition coefficients (LogK_ow_), which range from 4.01 to 8.67 [47,48]. This property strongly influences their environmental fate, leading to significant association with particles and sediments upon release into aquatic systems [24]. The spatial distribution of SCCPs in water is closely linked to anthropogenic activities such as industrial discharges, sewage treatment plant (STP) effluents, and general human influence, similar to the distribution patterns detected in soil [61,62,63,64,65,66]. It should be noted that monitoring data from Chinese aquatic environments reveal substantial SCCP contamination. SCCP concentrations in surface waters across China obviously range from 4.10 to 65,640 ng/L, with significantly higher levels distinguished in industrialized regions and areas influenced by e-waste recycling activities, as listed in Table 3. Quantitatively, SCCP concentrations in Chinese surface waters (4.1–65,640 ng/L) are generally higher than those reported in Japan (16–360 ng/L) [67], France (50–4000 ng/L) [68], and North America (0.606–1.935 ng/L) [69], but are comparable to those found in industrial regions of the United Kingdom (2000–8000 ng/L) [70]. Industrial wastewater has been identified as a crucial source of SCCPs entering STPs, with the subsequent discharge of effluents driving their occurrence in receiving waters [71].

As shown in Table 3, a distinct spatial gradient of SCCPs is evident within China’s marine waters. SCCP concentrations in the Yellow and Bohai Seas seem to be higher than those in the South China Sea [56,72]. Furthermore, lower levels detected in the central Bohai Sea and Liaodong Bay suggest that SCCPs in this basin likely originate from substantial riverine inputs, particularly along the Shandong Peninsula [31,73]. This pronounced spatial gradient in China indicates higher concentrations in eastern and coastal regions compared to western and inland areas [73,74]. Moreover, urban waterways and regions receiving industrial effluents consistently exhibit higher contamination levels than rural or background sites. However, a comprehensive and robust understanding of the precise spatial dynamics of SCCPs in aquatic systems remains limited by the availability of extensive, high-resolution monitoring data. Future research efforts should prioritize expanding the spatial and temporal scope of sampling to better elucidate sources, transport pathways, and sinks. The SCCP concentrations detected in Chinese freshwater systems tend to be higher than those reported in Japan, France, and North America, indicating the influence of industrial discharge and wastewater treatment limitations. This status reflects the strong relationship between local industrial activities and aquatic contamination in China.

**Table 3:** SCCP concentrations in Chinese water samples.

Region	Site	Sample	Concentration (ng/L)	Detection Method	Sampling Time	Ref.
Eastern China	Shanghai	River water	15.0–1640	GC-ECNI-MS	2016	[75]
	The intertidal zone of the Shandong Peninsula	Seawater	370–548 (Yellow Sea); 573–1978 (Bohai Sea)	HRGC-ECNI-LRMS	2017	[76]
	Xiaoqing River	River water	7.4–470	GC/NCI-MS	2014	[62]
Southern China	Pearl River Estuary	Seawater	180–460	GC-ECNI-LRMS	2012–2013	[72]
	An enclosed freshwater pond	Pond water	61.0 ± 5.50	GC-ECNI-MS	2014	[77]
Northern China	Sewage treatment plant	Wastewater	27.0–184	HRGC-ECNI-LRMS	2012	[78]
	Beijing	Drinking water	20.0–26.0	GC-TOF-HRMS	2016	[46]
	Baiyangdian Lake	Lake water	1563–56,306	GC-MS	2016	[79]
Northeastern China	Liaodong Bay	Seawater	4.10–13.1	GC-ECNI-MS	2012	[73]
	Pulandian Bay	Seawater	494–1490	GC-ECNI-MS	2012	[80]
Central China	The middle reaches of the Yangtze River	River water	1131–65,640	GC-MS	2016	[79]
China sea regions	Bohai Sea	Seawater	11.0–110.0	GC-QTOF/NCI-HRMS	2016	[31]
	East China Sea	Seawater	12.2–430	GC-ECNI-MS	2019	[74]

### 2.3. Soil

Soil acts as a reservoir for SCCPs, which originate from the long-distance transport and enter soil via multiple pathways under certain conditions and anthropogenic activities, such as atmospheric deposition, wastewater irrigation, and industrial discharges, and relative SCCP concentrations are confirmed in these regions [23,24,25]. As listed in Table 4, SCCP concentrations in soil across China exhibit a range of ND-554,161 ng/g dw, with the highest levels observed in industrial soils. These levels substantially exceed those reported in European countries (0.6–570 ng/g dw) [81,82] and Antarctica (3.5–32.8 ng/g dw) [83]. Major SCCP contamination hotspots are strongly linked to specific human activities, notably e-waste dismantling, industrial manufacturing, and areas influenced by sewage treatment plants. Furthermore, agricultural soils typically exhibit higher SCCP concentrations than ambient soils, and urban areas show higher levels than rural regions, suggesting significant contributions from practices such as wastewater irrigation and sewage sludge application.

The distribution of SCCPs in soil is analogous to that of gaseous SCCPs, with concentrations in northern China exceeding those in eastern, northeastern, and southern regions (Table 4), indicating that atmospheric deposition is a dominant input pathway of SCCPs in soils. Surprisingly, higher SCCP levels can be found in soils from the Yunnan province, located on the southwest border of China, attributed to a combination of local human activities and atmospheric transport [84]. Similarly, detectable SCCP levels on the remote Tibetan Plateau, a high-altitude background region with minimal local emission sources, highlight the role of long-range atmospheric transport [85,86]. This transport and subsequent deposition are amplified in high-altitude regions by the “mountain cold-trapping” effect, where low temperatures and high precipitation promote the condensation and sequestration of SCCPs from the atmosphere [87].

**Table 4:** SCCP concentrations in Chinese soil samples.

Region	Site	Sample	Concentration (ng/g dw)	Detection Method	Sampling Time	Ref.
China	31 provinces of China	Agricultural soil	38.7–1609	GC-ECNI-LRMS	2016	[88]
Eastern China	Shanghai	Ambient soil (background)	0.42–420	GC-MS	2011	[89]
	Shanghai	Ambient soil (urban)	ND-615	GC-MS	2011	[90]
	Shanghai	Ambient soil (suburban)	ND-679	GC-ECNI-MS	2011	[91]
	Shanghai	Agricultural and industrial surface soils	52.6–237.6 (agricultural); 98.3–977.1 (industrial)	GC-ECNI-LRMS	2019–2021	[92]
	An e-waste dismantling area in Taizhou	Ambient soil	226–755	HRGC-ECNI-LRMS	2011	[93]
	A CP production plant in Zibo	Ambient and industrial soil	27,508–554,161 (in plant); 102–441 (surrounding environment)	GC-ECNI-MS	2016	[35]
	An e-waste dismantling area in Taizhou	Ambient and agricultural soil	68.5–220,000	GC × GC-ECNI-MS	2017	[94]
	The intertidal zone of the Shandong Peninsula	Ambient soil	50.1–266	HRGC-ECNI-LRMS	2017	[76]
	Liaocheng city of Shandong province	Surface farmland soil	5.41–381	GC-QTOF-NCI-MS	2017	[95]
	Yangkou chemical industrial park in Jiangsu province	Ambient and industrial soil	37.5–996	GC × GC-ECNI-MS	2018	[61]
	Zhoushan	Surface soil samples collected from the contaminated area	72–3842	GC × GC-ECNI-MS	2018–2019	[55]
Southern China	Pearl River Delta	Ambient soil	1.90–236	GC-ECNI-LRMS	2009–2010	[39]
	Guangzhou	Ambient and agricultural soil	1.45–25.5	GC-ECNI-MS	2009–2010	[96]
	Guangzhou	Ambient and agricultural soil	6.80–541	GC-ECNI-MS	2012	[97]
	A CP production plant brownfield site in Guangzhou	Industrial soil	ND-5090	HPLC-ESI-QTOF-MS	2018	[98]
	Jiangmen	Electronic industrial park soil	144.4–1160	GC-ECNI-LRMS	2025	[99]
Southwestern China	Chengdu	Ambient and agricultural soil	0.22–3.26	GC-ECNI	2014	[100]
	Yunnan	Ambient soil	79.0–948	GC × GC-ECNI-MS	2016	[84]
Northern China	Beijing	Agricultural soil	160–1450	GC-ECNI-LRMS	2010	[101]
	Factories in a non-ferrous metal recycling park located in Hebei	Surface soil	121–5159	GC × GC-ECNI-MS	2019	[102]
Northeastern China	A CP production plant in Dalian	Ambient and industrial soil	1018–1824 (in plant); 24.8–482 (surrounding environment)	GC-ECNI-MS	2013–2014	[65]
Background region	Tibetan Plateau	Ambient soil	81.6 ± 31.1	GC-ECNI-MS	2012–2014	[85]
	Tibetan Plateau	Soils from the urban landfill and rural dumpsites	56.8–1348	GC-ECNI-MS	2017	[103]

### 2.4. Sediment

Due to their resistance to biodegradation and high hydrophobicity, SCCPs readily accumulate in sediments, posing a persistent risk to aquatic ecosystems and human health [104]. SCCP contamination in Chinese sediments is pronounced, exhibiting remarkable spatial patterns driven by anthropogenic inputs and environmental transport processes. As summarized in Table 5, sediment monitoring data indicate that SCCP concentrations across China range from ND to 397,600 ng/g dw, with the highest level found in contaminated site sediments (8.3–397,600 ng/g dw). These values significantly exceed those reported for Japan (1.3–27.4 ng/g dw) [105], North America (147–410 ng/g dw) [106,107], and several European countries (2–800 ng/g dw) [108,109,110,111]. However, SCCP concentrations are comparable to levels found in industrial areas of the UK [70] and Spain [112,113]. Significant point sources include freshwater sediments in e-waste processing zones and areas receiving discharges from sewage treatment plants (STPs) [71].

Similar to patterns observed in water and air, SCCP levels in sediments decrease from the coast to offshore areas. Nationally, concentrations generally follow the order: northern > southern > northeastern > central > eastern > southwestern > northwestern regions. This variation in SCCP concentrations is further influenced by habitat type, with levels typically higher in freshwater sediments than in marine sediments, consistent with global trends [67,105]. The highest SCCP concentrations are found in estuarine and coastal sediments [105,114,115,116], with levels declining both offshore and with sediment depth [117,118], strongly implicating riverine input as the primary source of marine sediments. Background sites exhibit minimal contamination, with the lowest levels detected in Qinghai Lake on the Tibetan Plateau [119]. The observed distribution is primarily governed by the intensity of local human activities coupled with hydrological and atmospheric transport. Relative SCCP concentrations in sediments near population and industrial centers result from high-emission sources, with SCCPs and MCCPs released into adjacent rivers via wastewater overflow and surface runoff. Long-range atmospheric transport contributes to background contamination even in remote regions, while coastal deposition is dominated by riverine plume dispersal.

The exceptionally high SCCP levels in Chinese soils and sediments, especially near e-waste dismantling and industrial zones, distinguish China from most developed regions, where stricter regulations have reduced environmental accumulation.

**Table 5:** SCCP concentrations in Chinese sediment samples.

Region	Site	Sample	Concentration (ng/g dw)	Detection Method	Sampling Time	Ref.
China	China Coastal Estuaries	Marine sediment	242–1450	GC-ECNI-MS	2011	[80]
	Nine lakes	Lake sediment	59.0–650	ESI-QTOF-MS	2013–2019	[119]
	Urban black odorous rivers flowing through 73 cities	River sediment	8.3–94,000	GC-ECNI-MS	2018	[120]
Eastern China	Shanghai	River sediment	ND-2020	GC-ECNI-MS	2016	[75]
	Laizhou Bay	River and marine sediment	8.40–2000 (river sediment); 5.10–22.0 (marine sediment)	HRGC-ECNI-LRMS	2016	[121]
	The intertidal zone of the Shandong Peninsula	Marine sediment	17.6–453	HRGC-ECNI-LRMS	2017	[76]
	Jiaojiang River in Taizhou	River sediment	32.5–12,900	HRGC-ECNI-LRMS	2017	[94]
	Xiaoqing River in relation to the Laizhou Bay environment	River sediment	9.1–16,000	GC-ECNI-MS	2014	[62]
Southern China	Pearl River Delta	River sediment	224–3800	GC-ECNI-LRMS	2009–2010	[122]
	PRD, Shenzhen, and Hong Kong	Marine sediment	ND-1540	HRGC-ECNI-LRMS	2012–2013	[105]
	Pearl River Estuary	Marine sediment	180–620	GC-ECNI-LRMS	2012–2013	[116]
	Longtang, Qingyuan	Pond sediment	3200–13,700	GC-MS	2010	[123]
	An enclosed freshwater pond	Pond sediment	82.0–350,000	GC-ECNI-MS	2014	[77]
Central China	Henan section of the Yellow River	River sediment	11.8–2792	GC-ECNI-LRMS	2014	[124]
	The middle reaches of the Yellow River	River sediment	11.6–9760	GC × GC-TOFMS	2015	[125]
	The middle reaches of the Yangtze River	River sediment	4.19–41.6	GC × GC-TOFMS	2015	[126]
	The middle reaches of the Yangtze River	River sediment	251.9–397,600	GC-MS	2015	[79]
Northern China	Beijing	Lake sediment	1100–8700	GC-ECNI-LRMS	2010	[71]
	Haihe River Basin	River sediment	131.83–1767.71	GC × GC-TOFMS	2021	[127]
Northeastern China	Liaodong Bay	Marine sediment	65.0–541	GC-ECNI-MS	2012	[73]
	Liaohe Estuary	Marine sediment	64.9–1683	GC-ECNI-MS	2010	[128]
China Sea areas	Bohai Sea	Marine sediment	97.4–1757	GC-ECNI-MS	2010	[114]
	East China Sea	Marine sediment	9.38–41.6	NA	2012	[129]
	East China Sea	Marine sediment	89.6–351	GC-ECNI-MS	2019	[74]

### 2.5. Biota

The widespread environmental presence, persistence, and lipophilicity of SCCPs facilitate their bioaccumulation and potential biomagnification within organisms, posing significant risks to ecological health and human exposure. Monitoring data in Table 6 confirm the widespread occurrence of SCCPs across diverse food chains, showing that SCCP concentrations in biota range from ND to 30,000 ng/g dw, with the highest level found in freshwater organisms (3.90–30,000 ng/g dw). On average, concentrations are higher in aquatic than in terrestrial organisms. Within aquatic systems, freshwater species typically exhibit higher burdens than their marine counterparts, reflecting the distribution trend observed in sediments. Several studies have elucidated biomagnification potential across various food webs, including estuarine, lacustrine, and remote polar ecosystems. This process is influenced by factors such as the selective transfer of SCCP homologs, feeding ecology, and biotransformation rates within organisms.

Accumulation patterns of SCCPs are primarily governed by habitat and trophic ecology. In aquatic systems, invertebrates accumulate significant levels of SCCPs through direct sediment ingestion and filter feeding. This bioaccumulation at lower trophic levels leads to even higher concentrations in predatory fish, demonstrating trophic transfer [73,130,131]. For terrestrial organisms, particularly plants, uptake occurs primarily through roots and leaves from contaminated soil and air [86,132,133]. Notably, a negative correlation between SCCP concentration and trophic level has been observed in several food chains, suggesting that a trophic dilution effect may occur alongside the biomagnification potential for specific congeners [85].

The spatial distribution of SCCPs in biota across China reflects regional pollution patterns, with organismal burdens highest in eastern and northeastern regions, followed by northern and southern areas. Background sites, such as the Tibetan Plateau, exhibit concentrations one to two orders of magnitude lower than emission zones, yet these levels still substantially exceed those reported for polar regions [83], indicating measurable long-range atmospheric transport. Pollution hotspots are clearly identified, with the highest concentrations found in species such as catfish (11,400–70,400 ng/g dw) inhabiting areas near e-waste recycling sites [134] and in fish from major water supply sources like Dianshan Lake [135]. Consistently higher SCCP concentrations in organisms from estuarine regions compared to other coastal areas reveal the dominant role of riverine inputs from contaminated watersheds [72,116,128]. Significant data gaps remain for central, southwestern, and northwestern China, highlighting the need for expanded spatial monitoring to fully characterize national exposure profiles.

**Table 6:** Concentrations of SCCPs in biota samples in China (note: lw = liquid weight).

Region	Site	Sample	Concentration (ng/g dw)	Detection Method	Sampling Time	Ref.
Eastern China	An e-waste dismantling area in Taizhou	Snail	137–821	GC-ECNI-MS	2008 and 2010	[66]
	Yangtze River Delta	Snake	1900–19,000 (liver); 1900–22,000 (muscle)	APCI-QTOF-MS	2011	[136]
	Yangtze River Delta	Black-spotted frogs	ND-9200 ng/g lw	APCI-QTOF-MS	NA	[137]
	Dianshan Lake, Shanghai	Fish	810–30,000	GC-NICI-LRMS	2014	[135]
	Shanghai	Pine needle	ND-13,600	GC-NICI-MS	2015	[133]
	Yangtze River Delta	Wildlife species	69.0–360 (fish); 110–1400 (reptile); 710–3700	APCI-QTOF-MS	2017	[138]
	Crab farms in river basins along the Yangtze River	Chinese mitten crabs	82–1760 ng/g lw	GC × GC-MS/MS	2019	[139]
Southern China	An e-waste recycling site in Qingyuan	Terrestrial bird species	620–17,000	GC-ECNI-MS	2011–2012	[140]
	Hong Kong water	Marine organisms	15.3–569 (fish); 11.1–72.2 (crustacean)	HRGC-ECNI-LRMS	2012	[131]
	Pearl River Estuary	Marine biota	74.0–2000	GC-ECNI-MS	2012–2013	[116]
	Pearl River Estuary	Marine organisms	61.0–930	GC-ECNI-LRMS	2013	[72]
	An e-waste recycling site in Guiyu	Catfish and pigeon	11,400–70,400 (catfish); 4700–11,000 (pigeon)	GC-NCI-MS	2013	[134]
	An e-waste contaminated pond in Qingyuan	Aquatic organisms	1200–250,000 ng/g lw	GC-ECNI-MS	2016	[141]
Northern China	Beijing	Fish	1000–3500	HRGC-ECNI-LRMS	2010	[71]
	Beijing	Pine needle and bark	320–4270 (pine bark); 400–4010 (pine needle)	GC-ECNI-LRMS	2011	[132]
Northeastern China	Liaohe Estuary	Zooplankton, shellfish, shrimp, and fish	759–17,000	GC-ECNI-MS	2009	[80]
	Liaohe Estuary	Mollusk	1550–11,900	GC-ECNI-MS	2010	[128]
	Liaodong Bay	Organisms	1600–17,000	GC-ECNI-MS	2012	[73]
	A CP production plant in Dalian	Coniferous leaves	1281–2197 (in plant); 219–1742 (surrounding)	GC-ECNI-MS	2013–2014	[65]
	Liaodong Bay	Fish	374–8430	GC × GC-ECNI-HRTOF-MS	2014	[130]
China Sea areas	Bohai Sea	Mollusk	64.9–5510	GC-ECNI-LRMS	2009	[142]
	Bohai Sea	Bivalve	476–3270	GC-ECNI-MS	2010	[114]
	Bohai Sea	Mollusk	28.2–6026	GC-NCI-QTOF-LRMS	2011–2018	[143]
	East China Sea	Organisms	12.8–15.6 (Zooplankton); 31.0–1819 (Fish); 45.9–83.4 (Shrimp); 79.5–662 (Crab); 131–190 (Shellfish); 35.6–177 (Snail); 34.8–90.0 (Cephalopod);	GC-ECNI-MS	2019	[144]
	Yellow Sea (YS), East China Sea (ECS), and South China Sea (SCS)	Fish	13.5–60.0 (YS); 15.4–63.2 (ECS); 9.30–38.0 (SCS)	Stimulated	2008–2012	[145]
	South China Sea (Hong Kong)	Marine mammals	280–3900 (porpoises); 430–9100 (dolphins)	HRGC-ECNI-LRMS	2004–2014	[146]
	Nansha Islands of the South China Sea	Fish	37.9–25,400 ng/g lw	GC-ECNI-MS	2017	[147]
	South China Sea	Mussels, clams, giant tubeworms, slim tubeworms, shrimps, snails, sea cucumbers, brittle stars, and crabs	572.8–1943.1 ng/g lw (mussels); 169.3–674.2 ng/g lw (clams); 236.0–249.1 ng/g lw (giant tubeworms); 3134.8–4273.5 ng/g lw (slim tubeworms); 644.6–3244.0 ng/g lw (shrimps); 143.6–7189.5 ng/g lw (snails); 2985.3–4527.7 ng/g lw (sea cucumbers); 644.4–3179.2 ng/g lw (brittle stars)	UPLC-ESI-Orbitrap MS	2020–2022	[148]
Background area	Tibetan Plateau	Fish	3.9–107	HRGC-ECNI-LRMS	2007–2010	[149]
	Tibetan Plateau	Bark, needle, lichen, and moss	2900–7000 (bark); 2400–6400 (needle);	GC-QTOF-NCI-MS	2010–2016	[86]
	Tibetan Plateau	Plant, plateau pika, and eagle	1400–6100 (liche); 258 ± 126 (plateau pika)	GC-ECNI-MS	2012–2014	[85]

### 2.6. Food

Dietary intake is a major route of human exposure to SCCPs, as these pollutants accumulate in various foodstuffs at substantial concentrations. Due to their high lipophilicity and octanol–water partition coefficients (K_ow_), SCCPs tend to accumulate to a greater extent in animal-derived foods than in plant-based products [150,151,152,153]. As the world’s largest producer and consumer of SCCPs, China faces significant concerns regarding food contamination, prompting extensive research into their occurrence across the food supply. Accordingly, investigations have confirmed the widespread presence of SCCPs in diverse food categories, including eggs, meats, fishes, oils, potatoes, cereals, tea, legumes, and even baby food, as listed in Table 7. The studies summarized in Table 7 comprise 23 publications covering 18 different food types, which are classified into raw ingredients, processed foods, oils, and infant nutrition products. This section concludes the distribution of SCCPs in foods across China to establish a benchmark for future assessment and management.

According to the works listed in Table 7, a clear geographical trend is evident, with higher SCCP concentrations found in foods from eastern and southern China compared to central and western regions, indicating that pollution levels are positively correlated with regional industrial and anthropogenic activity. Concentration levels also vary markedly among food types. For instance, a study of 49 cooking oil samples detected SCCP homologs in 48 of them, with concentrations ranging from 9 to 7500 ng/g, leading to an estimated daily intake of 0.78–38 μg/d (based on a vegetable oil consumption rate of 32.7 g/d) [154]. Similarly, studies of 1710 grain samples from 19 Chinese provinces reported mean concentrations of 343 ng/g ww in grains and 328 ng/g ww in beans, which are generally lower than those in aquatic foods but higher than those in meats [155,156]. Notably, SCCP concentrations in infant foods (0.5–733 ng/g lw) are lower than in other food categories.

Dietary exposure assessments often employ the margin of exposure (MOE) approach, a quantitative tool utilized by scientific committees to evaluate the genotoxicity and carcinogenicity of chemical contaminants. The MOE below a defined threshold indicates a potential health concern [20]. While estimated daily intakes vary, averaging approximately 14.8 μg/day for residents of Beijing, current data suggest that SCCP levels in foods, including baby food, typically result in MOE values above 10,000 [154]. Although this indicates a low immediate risk based on traditional MOE thresholds, the pervasive presence of SCCPs in the food chain necessitates sustained monitoring and comprehensive risk evaluation to safeguard long-term public health.

**Table 7:** SCCP concentrations in food samples collected across China.

Food Type	Site	Concentration	Detection Method	Sampling Time	Ref.
Eggs	South China	64 ng/g ww	GC × GC-ECNI-MS	2020	[157]
	South China	46 ng/g ww	GC × GC-NCI-MS/MS	2022–2023	[158]
	Jinan, China	12.1–76.2 ng/g ww	APCI-qToF-MS	2020	[159]
	Rural Tibetan Plateau and Jiangxi province	MDL-42,900 ng/g ww (Tibetan Plateau); MDL-649 ng/g ww (Jiangxi province)	GC-QTOF-HRMS	2018–2021	[160]
Meats	20 provinces in China	129 ± 4.1 ng/g ww	GC × GC-ECNI-TOFMS	2011	[161]
	Beijing	117 ng/g ww	HRGC-ECNI-LRMS	2014–2016	[162]
	Jinan	132 ng/g ww	GC-ECNI-LRMS	2019	[151]
	Jinan	7.6–78.1 ng/g ww	APCI-qToF-MS	2020	[159]
	South China	69 ng/g ww	GC × GC-ECNI-MS	2020	[157]
	Takeout food online from restaurants in Beijing	248 ng/g ww	GC × GC-MS/MS	2022	[163]
Fishes	South China	55 ng/g ww	GC × GC-ECNI-MS	2020	[157]
	Jinan	10.6–123.2 ng/g ww	APCI-qToF-MS	2020	[159]
	Beijing	46 ng/g ww	HRGC-ECNI-LRMS	2014–2016	[162]
Aquatic foods and shellfishes	Beijing	60.5 ng/g ww	HRGC-ECNI-LRMS	2014–2016	[162]
	18 provinces in China	1472 ng/g ww	GC × GC-ECNI-TOFMS	2017	[164]
	Jinan, China	133 ng/g ww	GC-ECNI-MS	2019	[151]
	Jinan, China	10.6–123.2 ng/g ww	APCI-qToF-MS	2020	[159]
	South China	55 ng/g ww	GC × GC-ECNI-MS	2020	[157]
Oils	176 cooking oils and 19 oil containers collected from various markets in China	ND-16,055 ng/g	GC-QTOF-NCI-MS	2020	[165]
	Beijing, Fushun, Hong Kong, Shanghai, and Shenyang in China	<9–7500 ng/g	HRGC-ECNI-HRMS	2010, 2012	[154]
	Butter oil in Tibet, China	132 ng/g lipid	GC-QTOF-NCI-MS	2021	[166]
Cereals	19 provinces in China	343 ng/g ww	GC × GC-TOFMS	2011	[155]
	South China	17 ng/g ww	GC × GC-ECNI-MS	2020	[157]
	Jinan, China	38–207 ng/g ww	APCI-qToF-MS	2020	[159]
Tea	11 provinces in China	4.99–717 ng/g	GC × GC-ECNI-MS/MS	2020	[167]
Wine	China	ND-415 ng/mL	HPLC-ESI-Q-TOF/MS	2020	[168]
Noodles	China	1200 ng/g ww	GC × GC-ECNI-MS	2021	[153]
Starch	Takeout food online from restaurants in Beijing	77.2 ng/g ww	GC × GC-MS/MS	2022	[163]
Condiments	China	1400 ng/g ww	GC × GC-ECNI-MS	2021	[153]
Honey	North Beijing, China	37 ng/g	GC × GC-ECNI-MS	2022	[169]
Ready-made meals	Beijing, China	22.4–546 ng/g dw	GC-TOF-HRMS	2016	[46]
Vegetables	Beijing	11.7 ng/g ww	HRGC-ECNI-LRMS	2014–2016	[162]
	Jinan, China	16.7 ng/g ww	GC-ECNI-MS	2011	[151]
	Jinan, China	ND-4.9 ng/g ww	APCI-qToF-MS	2020	[159]
	South China	37 ng/g ww	GC × GC-ECNI-MS	2020	[157]
	Takeout food online from restaurants in Beijing	42.9 ng/g ww	GC × GC-MS/MS	2022	[163]
Fruits	Beijing, China	16.4 ng/g ww	HRGC-ECNI-LRMS	2014–2016	[162]
	Jinan, China	18.9 ng/g ww	GC-ECNI-MS	2011	[151]
	Jinan, China	7–29.3 ng/g ww	APCI-qToF-MS	2020	[159]
Legumes	19 provinces in China	328 ng/g ww	GC × GC-TOFMS	2011	[155]
	South China	17 ng/g ww	GC-ECNI-MS	2017–2018	[157]
Milk and dairy	5 provinces in China	750 ng/g lw	GC × GC-MS/MS	2018	[170]
	South China	38 ng/g ww	GC × GC-ECNI-MS	2020	[157]
	Jinan, China	19.4–173 ng/g ww	APCI-qToF-MS	2020	[159]
Baby food	12 provinces in China	681 ng/g lw	GC × GC-ECNI-HRTOFMS	2007	[171]
	16 provinces in China	733 ng/g lw	GC × GC-ECNI-HRTOFMS	2011	[171]
	16 provinces in China	303 ng/g lw	GC × GC-ECNI-HRTOFMS	2007, 2011	[172]
	Beijing, China	<0.5–54 ng/g lw	GC-ECNI-HRMS	2007–2010	[173]
	Shaoxing, China	37.9 ng/g lw	APCI-QTOF-HRMS	2010	[174]
	Jiaxing, China	28.6 ng/g lw	APCI-QTOF-HRMS	2015–2016	[174]
	Shanghai, China	35 ng/g lw	APCI-QTOF-HRMS	2015–2016	[174]
	Beijing, China	16.2–20.5 ng/g dw	GC-TOF-HRMS	2016	[46]
	China	6.22–273 ng/g	UPLC-Orbitrap-HRMS	2022	[175]

Overall, the distinct SCCP pollution patterns observed in China can be attributed to several country-specific reasons: (i) its dominant role as the global producer and consumer of CPs, (ii) the presence of e-waste recycling sites, (iii) high population density and rapid urbanization, and (iv) evolving but still developing regulatory frameworks. These factors collectively result in higher environmental concentrations, more complex exposure pathways, and greater uncertainty in risk assessment compared to other regions.

## 3. Identification and Characterization of SCCPs

Accurate SCCP quantification remains challenging due to isomeric complexity, matrix interference, and the lack of individual congener standards. This section focuses on three aspects relevant to laboratories and regulators: (1) extraction and cleanup techniques for different sample types, (2) instrumental methods (GC-ECNI-MS, GC × GC-TOF-MS, HRMS, etc.) employed for routine monitoring and research applications, and (3) key technical bottlenecks (e.g., differentiation of SCCPs from MCCPs, inter-laboratory variability) that still need to be addressed. Method performance characteristics are summarized in Table 8 and Table 9.

The concentration distribution of SCCPs varies greatly across environmental matrices, making extraction methods and the cleanup process particularly important. SCCP extraction from environmental samples commonly employs techniques such as accelerated solvent extraction (ASE), liquid–liquid extraction (LLE), and dispersive liquid–liquid microextraction (DLLME). Among these, ASE utilizes high temperature (50–200 °C) and pressure (100–3000 psi) for efficient automated extraction, offering advantages over LLE such as speed, reduced solvent use, and lower labor input. Post-extraction, a cleanup step is essential to purify the organic solvent extract by removing matrix interferences like fats and pigments, thereby simplifying the sample for subsequent instrumental analysis. Moreover, such cleanup can be used to eliminate other compounds chemically similar to CPs, such as PCBs, chlordane, toxaphene, etc. This process can also affect instrumental detection, thus improving the accuracy of quantitative methods [20]. After extraction and cleanup, instrumental methods are employed to identify the types and concentration levels of CPs in various environmental and biological samples. Because CPs are complex mixtures containing thousands of isomers, no single applicable CP standard can be used for semi-quantitative analysis of SCCPs, and only mixed CP standards are currently practicable [176,177]. For routine monitoring of food and human samples, GC-ECNI-MS with appropriate internal standards remains the most practical choice, offering acceptable sensitivity (LOD ~10–50 ng/g) and throughput (~20 samples/day), though inter-laboratory CVs of 20–40% remain a concern, as reported by Krätschmer et al. [178,179]. Current analytical measurement of SCCPs still requires further development, primarily due to the extreme complexity of SCCP mixtures, low chromatographic resolution between congeners, and difficulty in achieving precise quantification [180,181]. In this regard, our review provides a brief overview of the development of instrumental methods for environmental sample analysis between 2015 and 2025.

Based on the majority of literature reports (19 in total) between 2015 and 2025 (Table 8), sensitive and reliable analytical methods are essential for compliance monitoring of SCCPs in environmental matrices. The most widely employed techniques include one-dimensional gas chromatography (GC), comprehensive two-dimensional GC × GC equipped with electron capture negative ionization mass spectrometry (ECNI-MS), or time-of-flight MS (TOF-MS), and high-resolution GC–high-resolution MS (HRGC-HRMS). In addition, numerous optimization approaches exist to modify instrument sensitivity, including the selection of chromatographic column, MS source temperature, and internal standards [20]. Bogdal et al. presented an innovative method using a chlorine-enhanced atmospheric pressure chemical ionization (APCI) approach with a quadrupole TOF-HRMS (APCI-qTOF-HRMS) system for rapid quantification of CPs in environmental samples [180]. Among the diverse analytical methods, GC-ECNI-MS and GC × GC-based methods are commonly applied to analyze the environmental samples (water, sediment, and soil), with the latter offering improved separation for complex matrices. GC-MS-based methods with rigorous cleanup procedures are particularly suitable for food samples due to their high sensitivity and effective lipid removal. HRMS is increasingly preferred for human samples (blood, milk, tissues) because of the need for higher selectivity and lower detection limits [154,182,183,184,185]. Importantly, part of the variability distinguished in reported SCCP concentrations across different studies (as listed in Table 2, Table 3, Table 4, Table 5, Table 6 and Table 7) may be attributed to differences in analytical methodologies, including extraction efficiency, cleanup procedures, and instrumental techniques. This methodological variability should be taken into account when interpreting environmental and human exposure data. According to these reported studies, distinguishable differences in SCCP identification and quantification arise not only from the analytical instruments but also from sample preparation procedures. The typical characteristics of several main analytical methods are summarized in Table 9.

In conclusion, for routine monitoring of SCCPs in complex matrices, GC-ECNI-MS remains one of the most widely used and practical approaches due to its robustness, sensitivity, and relatively established protocols. Advanced techniques such as GC × GC-HRMS and Orbitrap-based methods provide superior resolution and congener-level information, but their application is currently limited by instrument complexity, data processing demands, and lack of standardized workflows. Therefore, a tiered analytical strategy may be appropriate where conventional GC-MS-based methods are employed for routine monitoring, while high-resolution techniques are reserved for detailed characterization and research purposes.

**Table 8:** Analytical procedures of SCCP determination taken into consideration in these reported works.

No.	Sample Preparation Procedure	SCCP Determination Method	Sample Type	Year	Ref.
1	ASE	GC-NCI-qTOF-HRMS	Air samples from Shergyla Mountain, southeast of the Tibetan Plateau (China)	2016	[182]
2	SE-MCC	HRGC-EI/HRMS	Sediment and biological samples from the Liaohe River Basin (China)	2016	[183]
3	ASE	GC × GC-ECNI-HRTOF-MS	Sediment samples from the middle reaches of the Yellow River, and fish samples from Bohai Bay (China)	2016	[186]
4	VALLME	GC-ECNI-MS	Sediments from the wastewater treatment plant and Lao-Jie River in northern Taiwan	2016	[187]
5	SE-MCC	APCI-QTOF-MS	Fish and sediment from Sweden	2017	[188]
6	SBSE-SCC	TD-GC-QqQ-MS/MS	Solution obtained from Dr. Ehrenstorfer (Germany)	2017	[189]
7	NA	UPLC-ESI-QTOF-MS	Human blood samples from fifty adult volunteers ranging in age from 24 to 45 years (China)	2017	[190]
8	ASE-MCC	GC × GC-LRMS	Commercial CP products from three factories and air samples from Beijing (China)	2018	[191]
9	HS-SPME	GC × GC-TOF-MS	Water samples from different sites in Beijing (China)	2018	[192]
10	SPE	GC-ENCI-MS	Water samples from different sites and effluents from the Gaobeidian wastewater treatment plant in Beijing (China)	2018	[193]
11	ASE	MALDI-TOF-MS	Indoor dust samples from Beijing (China)	2018	[194]
12	ASE-MCC	GC × GC-TOF-MS	Cereal samples and legume samples from 19 Chinese provinces (China)	2019	[155]
13	NA	LC/ESI-HRMS	Lard samples from regular market food (Germany)	2020	[195]
14	dSPE	GC-ECNI/MS	Food samples and lard samples from a previous European Union Reference Laboratory	2020	[196]
15	PLE	LC-ESI-HRMS	Fish samples from the pool (France)	2020	[197]
16	NA	LC-ESI-MSMS	Mixed plastic wastes from seven industrial waste-processing facilities in Japan (Japan)	2020	[198]
17	NA	GC × GC-ECNI-HRTOF-MS	CP commercial product samples from 150 manufacturers in east and northeast China (China)	2021	[184]
18	PLE	GC-APPI-HRMS	Fish samples (salmon was of aquaculture origin, tuna was caught in the Mediterranean Sea) (Spain)	2021	[199]
19	USE-SBSE	GC-QqQ-MS-MS	Purified water reservoir and the Danube River freshwater Sediment samples (Slovak Republic)	2021	[200]

Acronyms appear in the table: ASE—accelerated solvent extraction; dSPE—dispersive solid-phase extraction; MALD—-matrix-assisted laser desorption/ionization; PLE—pressurized liquid extraction; ECNI—electron capture negative ionization; ESI—electron spray ionization; HRGC—high-resolution gas chromatography; VALLME—vortex-assisted liquid–liquid microextraction procedure; LRMS—low-resolution mass spectrometry; MCC—multilayer cleanup column; QTOF—quadrupole time-of-flight; SBSE—stir bar sorptive extraction; SCC—single-layer cleanup column; TD—thermal desorption; SE—Soxhlet extraction; SPE—solid-phase extraction; HS-SPME—solid-phase microextraction in headspace mode; UPLC—ultrahigh-pressure liquid chromatography; USE—ultrasonic solvent extraction.

**Table 9:** Comparison and application fields of the main analytical technologies of SCCPs.

Techniques	Outstanding Advantages	Limitations	Applicable Fields
GC × GC-MS	Low quantitative interference from overlapping *m/z*; high resolution for structurally similar compounds	Prolonged analysis time; requires optimization of column systems and temperature programs; complex data processing	Precise separation and quantification of SCCP/MCCP isomers in environmental samples
GC-NCI-LRMS	High sensitivity and low cost; ECNI mode generates minimal fragments, suitable for routine analysis	Response factors influenced by chlorination degree, leading to potential quantitative bias; susceptible to homolog interference	Routine detection of low-complexity samples (e.g., industrial products) with limited budgets
GC/ECNI-QTOF-MS	High resolution for distinguishing SCCPs/MCCPs; strong resistance to matrix interference	Limited capability for LCCP analysis; requires complex sample pretreatment	Analysis of SCCPs/MCCPs in environmental samples (e.g., sediments, biological tissues)
GC/ECNI-Orbitrap-MS	Excellent compatibility with gas-phase systems; resolution (120,000) suitable for highly chlorinated CPs	Limited scanning range (*m/z* 250–810); restricted applicability for long-chain CPs	Quantitative analysis of highly chlorinated CPs (e.g., flame retardants)
LC/ESI-QTOF-MS	Compatibility with multiple CP types (SCCPs, MCCPs, LCCPs); reduced ion source loss	Low sensitivity for SCCPs; ionization efficiency affected by mobile-phase selection	Simultaneous detection of multiple CP types (e.g., consumer products, food contact materials)
LC/ESI-Orbitrap-MS	Ultrahigh resolution (140,000); precise differentiation of chain lengths and chlorination levels	Strict requirements for Mobile-phase purity; high instrument cost and operational complexity	Accurate identification of trace CPs in complex matrices (e.g., wastewater, sludge)

## 4. Human Body Exposure to SCCPs

This section reviews biomonitoring studies that measured SCCPs in Chinese populations. We organize the analysis by biological matrix (placenta (Section 4.1), breast milk (Section 4.2), blood (Section 4.3), and other tissues/organs (Section 4.4)) because each matrix provides different information regarding exposure timing (prenatal vs. postnatal), internal dose, and potential health relevance.

Dietary exposure, discussed in relation to food intake as mentioned in Section 2.6, is a primary exposure route for SCCPs, and SCCPs in the human body, including placenta, breast milk, blood, serum, and human tissues or organs, are classified as internal exposure [24]. The widespread use and extensive environmental detection of SCCPs have accelerated growing concern about internal exposure in humans. In this review, 20 biomonitoring studies published between 2015 and 2025 from various regions and time periods in China are listed in Table 10. Studies reporting SCCP concentrations in human matrices use diverse units: ng/g lw, ng/g ww, ng/g dw, or ng/mL (serum/plasma). Direct comparisons between studies using different units are problematic because SCCPs are highly lipophilic and lipid content varies dramatically across matrices. Therefore, cross-study comparisons based on different units are not directly made in this section.

Previous studies have shown that the fetus may be exposed to CPs during pregnancy. Research has proven the presence of SCCPs in maternal serum (15.9–584 ng/mL), placenta (10.2–132 ng/g tissue), and cord serum (8.46–223 ng/mL), confirming significant transfer from maternal serum to cord serum [201]. To date, studies on the human body burden of SCCPs remain remarkably insufficient due to difficulties in identifying and quantifying CP mixtures. With continued progress in characterization and analytic strategies, renewed scientific focus has been directed toward precisely elucidating the distribution of SCCPs within the human body, including placenta, breast milk, blood, and organs (fat, kidney, liver, brain, bone, etc.). According to currently published data, the body burden of CPs in China is likely higher relative to that in other countries and regions.

**Table 10:** Concentrations of SCCPs in human body samples in China.

Sample	Site	Concentration	Unit	Detection Method	Sampling Time	Ref.
Human placenta	China	<36.8–782.1	ng/g dw	GC-ECNI-LRMS and GC-QTOF-HRMS	NA	[202]
	Henan province	98.5–3771	ng/g lw	GC-QTOF-HRMS	2016	[203]
	Wuhan	10.2–132	ng/g ww	GC-ECNI-LRMS	2015–2016	[201]
	Guangzhou	249–691	ng/g lw	GC-ECNI-LRMS	2016–2017	[204]
	Mianyang	14.3–108	ng/g ww	GC-ECNI-LRMS	2018	[205]
Breast milk	Beijing	<20.0–54.0	ng/g lw	HRGC-ECNI-HRMS	2007–2010	[173]
	Shijiazhuang	210–16,120	ng/g lw	GPC-GC-ENCI/MS	2014–2015	[206]
	Rural China	68.0–1580 (2007); 65.6–2310 (2011)	ng/g lw	GC × GC-ECNI-HRTOFMS	2007 and 2011	[172]
	Urban areas in China	170–6150 (2007); 131–16,100 (2011)	ng/g lw	GC × GC-ECNI-HRTOFMS	2007 and 2011	[171]
	3 cities in the Yangtze River Delta (Shanghai, Jiaxing, and Shaoxing)	<LOD-676	ng/g lw	APCI-QTOF-HRMS	2010–2016	[174]
	Shanghai	771	ng/g lw	GC × GC-orbitrap-HRMS	2016–2017	[207]
	Urban and rural areas in China	131–808 (urban areas); 139–1543 (rural areas)	ng/g lw	GC × GC-ECNI-MS	2017	[208]
	Mianyang	29.2–271	ng/ML	GC-ECNI-LRMS	2018	[205]
Human blood	Shenzhen	370–35,000	ng/g lw	UPLC-QTOFMS	2012	[190]
	Beijing	2570–57,800 (maternal serum); 3750–40,500 (cord serum)	ng/g lw	GC × GC-TOFMS	2013	[209]
	Dalian	<MDL-203 (Human plasma)	ng/g ww	HRGC-ECNI-LRMS	2015	[210]
	Wuhan	15.9–584 (maternal serum); 8.46–223 (cord serum)	ng/ML	GC-ECNI-LRMS	2015–2016	[201]
	Hangzhou	206–1448 (serum)	ng/g lw	GC-NCI-MS	2016–2018	[211]
	Guangzhou	407–1570 (Maternal plasma); 499–1830 (Maternal RBCs); 376–1660 (Cord plasma); 520–1780 (Cord RBCs)	ng/g lw	GC-ECNI-LRMS	2016–2017	[204]
	Guangzhou	1.00–5.45 (human serum)	ng/ML	HPLC-ESI-Q-TOF/MS	2018	[212]
	Mianyang	51.0–620 (maternal serum); 13.3–242 (cord serum)	ng/ML	GC-ECNI-LRMS	2018	[205]
	Jinan	1670–42,700 (serum)	ng/g lw	ULPC-qTOF-HRMS	2019	[213]
	Jinan	1320–24,100 (serum)	ng/g lw	APCI-qTOF-MS	2020	[214]
	Guangzhou	4.55–37.7 (serum)	ng/ML ww	UHPLC-Orbitrap-HRMS	2015–2016	[215]
Human tissues or organs	Northern China	19.2–877 (hair); 57.7–355 (Nail)	ng/g dw	GC-QTOF-HRMS	2018	[216]

LOD—Limit of detection. MDL—Method detection limit. RBCs—Red blood cells.

### 4.1. SCCPs in Human Placenta

A growing body of research, summarized in Table 10, has verified the widespread occurrence and placental transfer of CPs by analyzing matched maternal serum, placental, and umbilical cord serum samples. These studies are associated with fetal exposure during pregnancy. CPs, including both SCCPs and MCCPs, have been detected in 100% of analyzed placenta and cord serum samples from multiple Chinese cities (Mianyang, Beijing, and Wuhan) [201,205,209], suggesting CP exposure to fetuses during pregnancy. During placental transport, passive diffusion is identified as the primary transfer pathway [209], although the placenta partially retains CPs, thereby modulating fetal exposure [205]. Moreover, comparative analysis means that SCCPs have a greater transplacental transfer potential than MCCPs, as evidenced by higher placenta-to-maternal serum concentration ratios, suggesting that they may pose a more significant direct risk to the fetus [201]. Concentrations of SCCPs in human placenta also exhibit notable geographical variation. For instance, SCCP levels in placentas from the Henan province (98.5–3771 ng/g lw) were observed to be higher than those reported from other regions in China [203]. This disparity, along with the near-universal finding of higher SCCP concentrations in maternal blood than in placental tissue (Table 10), highlights the role of external exposure factors. Maternal exposure is likely influenced by a combination of local environmental pollution, occupational settings, dietary habits, and residential characteristics, although the precise contribution of each factor requires further elucidation. Therefore, current findings establish clear evidence of exposure but highlight the need for more robust epidemiological data. Future studies should prioritize larger, demographically diverse cohorts to accurately determine population-level body burdens, clarify dose–response relationships, and investigate the long-term health implications of prenatal SCCP exposure.

### 4.2. SCCPs in Breast Milk

Research from multiple Chinese groups has consistently confirmed the presence of SCCPs in breast milk (Table 10), establishing it as a significant exposure pathway for infants [206]. The efficiency of SCCP transfer into milk is influenced by their physicochemical properties, with higher transfer potential observed for congeners having lower chlorination degrees and shorter carbon-chain lengths. As listed in Table 10, reported concentrations in Chinese breast milk tend to be higher than those from Japan, Korea, Norway, and Sweden [173,174]. A distinct urban–rural gradient has been observed, though specific concentration comparisons require careful interpretation of cited values [171,172]. Monitoring data indicate a sharp decrease in SCCP concentrations in both urban and rural areas from 2007 to 2017 [171,172,208]. Infant exposure is dynamic, with a calculated average daily intake via breast milk decreasing from 13.0 μg/kg/day at 1 month to 2.5 μg/kg/day by 6 months of age. Furthermore, SCCP exposure concentrations during lactation have been reported to be over 100 times higher than during gestation [205], revealing this postnatal window as critical for risk assessment. Maternal factors also play a role, as studies report a positive correlation between SCCP concentrations in breast milk and maternal body weight at the end of gestation [206].

Margin of exposure (MOE) values used in this review are derived from toxicological reference points, typically no-observed-adverse-effect levels (NOAELs) obtained from animal studies. These endpoints include liver toxicity (e.g., hepatocellular changes), thyroid effects, and developmental toxicity. In general, an MOE value greater than 1000 is considered to indicate low concern for human health when extrapolating from animal data, as it incorporates uncertainty factors accounting for interspecies differences and human variability. Although SCCP concentrations in food and human matrices in China are relatively high compared to some other regions, MOE-based assessments suggest that current exposure levels remain substantially below thresholds associated with adverse effects. Such an obvious discrepancy appears because MOE reflects the margin between estimated human exposure levels and experimentally derived toxicological thresholds, rather than absolute exposure concentrations, and therefore is most relevant for assessing immediate or short-term risk. Meanwhile, relatively high environmental levels do not necessarily translate into immediate health risks if exposure remains far below toxicological thresholds. However, these assessments do not fully capture longer-term risks associated with the persistence and bioaccumulation of SCCPs. Due to their environmental stability, SCCPs can accumulate in ecosystems and biota over time, potentially leading to increased exposure levels in the future. Furthermore, vulnerable populations such as infants, pregnant women, and individuals with higher dietary intake may experience different exposure levels and sensitivities, which are not fully represented in generalized risk assessments. Overall, while current MOE-based assessments suggest low immediate risk, these findings should not be interpreted as an absence of concern, inferring further concern about the importance of continuous monitoring and refinement of risk assessment frameworks, particularly in regions with elevated exposure levels.

### 4.3. SCCPs in Human Blood

SCCPs have been detected in human blood samples worldwide, with reported concentrations covering a wide range (Table 10). Research has primarily focused on maternal and cord serum, with occasional analysis of red blood cells. A consistent finding across studies is that SCCP concentrations in cord serum are approximately one-third lower than in paired maternal serum (Table 10), suggesting possible transplacental transfer from mother to fetus. It should be noted that the SCCP concentrations identified in human blood samples from China appear to be higher than those reported in some other countries [3]; however, direct comparison is complicated by differences in analytical methods and study design. Such an observation is consistent with China’s dominant role in the global production and consumption of SCCPs in recent decades, revealing an association between regional industrial activity and population exposure [4]. The general trend of elevated SCCP exposure in China exhibits regional heterogeneity. For example, a study in Hangzhou (2016–2018) found significantly lower blood levels compared to other Chinese regions. Zhang et al. [211] suggest that this discrepancy may be attributed to local factors, including unique demographic and socioeconomic profiles, specific exposure scenarios, and dietary habits. However, the authors notice that these findings are tempered by several limitations, such as potential sampling issues and a lack of data on local SCCP production profiles, highlighting the need for more refined exposure assessment methodologies.

### 4.4. SCCPs in Human Tissues or Organs

Globally, research on the distribution of SCCPs in human tissues and organs beyond blood, milk, and placenta remains very limited, with only a handful of studies conducted to date (Table 10). Three pioneering works have examined SCCPs in specific tissues [216,217,218]. An early study by Campbell and McConnell (1980) detected CPs (C_10–30_) in the fat, liver, kidney, and brain of 24 individuals from the UK (ages 20 h to 92 years), confirming the highest concentrations in fat and liver [217]. Later, Müller and Schmid analyzed CPs in the adipose tissue of a 65-year-old Swiss male using GC-MS analysis [218]. More recently, Han et al. provided the first report of SCCPs and MCCPs in human hair and nails from northern China, suggesting their potential for non-invasive biomonitoring while noting the need to establish correlations with internal tissues and health effects [216].

The reported concentration ranges of SCCPs in major human matrices from China are as follows: placenta, ND-3771 ng/g lw [203]; breast milk, ND-16,100 ng/g lw [206]; blood, 206–57,800 ng/g lw [209]; and other tissues, 19.2–877 ng/g dw [216]. Data on SCCPs in human hair and nails remained scarce, with only one study reporting their levels in these matrices. No distinct regional variation in SCCP profiles within the human body has been identified (Table 10), which is consistent with diet being a primary exposure pathway. Additionally, compared to other countries, the observed high SCCP levels in Chinese blood and breast milk samples may partly reflect a concentration of research activity in China and other high-income Northern Hemisphere countries, limiting global population representativeness. Therefore, expanding studies to diverse geographical and demographic groups is essential. Notably, the frequent use of pooled samples, often necessary due to analytical sensitivity requirements, obscures inter-individual variation and prevents the investigation of correlations between specific SCCP levels and individual health risks. It is important to note that most of the available human studies are observational and cross-sectional in nature. Therefore, causal relationships between SCCP exposure and health outcomes cannot be established. Potential factors, including co-exposure to other environmental contaminants, lifestyle variables, and socioeconomic differences, may influence the observed associations. In addition, limitations such as relatively small sample sizes and the use of pooled samples in some studies further restrict the strength of the conclusions. Consequently, the current evidence should be interpreted with caution. Moreover, future research should prioritize longitudinal cohort studies and mechanistic investigations to better elucidate causal relationships between SCCP exposure and adverse health outcomes.

## 5. Toxicity, Toxicokinetics, and Adverse Health Effects of SCCPs in Humans

This section critically evaluates the evidence for SCCP toxicity, distinguishing prominent findings from animal and in vitro studies from emerging human association studies. We first summarize organ-specific toxicity (liver, kidney, thyroid) and systemic effects (developmental, neurotoxic, hematological, metabolic) in Section 5.1, then review toxicokinetic data (absorption, distribution, metabolism, excretion) in Section 5.2, and finally examine human health effects (hepatic diseases, thyroid dysfunction, diabetes, etc.) in Section 5.3. A key theme throughout is the distinction between rodent-specific mechanisms (e.g., male rat α2u-globulin nephropathy) and pathways relevant to human risk assessment.

### 5.1. Toxicity of SCCPs

**Figure 2:**
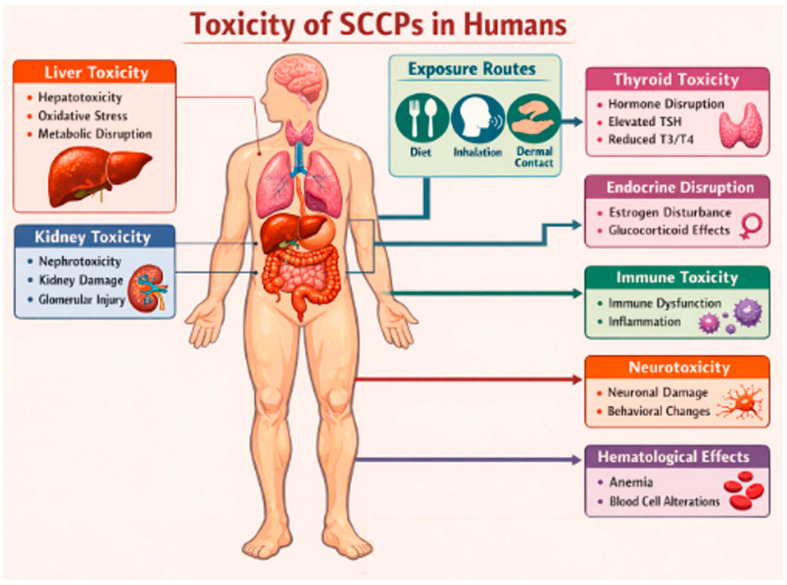
Toxicity and adverse health effects of SCCPs in humans.

To date, research on SCCPs has predominantly focused on their environmental occurrence and distribution, while investigations into their toxicological effects and the underlying molecular mechanisms remain insufficient. Evidence for SCCP toxicity is derived from multiple levels, including in vitro studies, animal experiments, and human epidemiological investigations, each with different strengths and limitations. Early toxicological assessments, primarily based on high-dose in vitro and animal studies, concluded that SCCPs exhibit low acute toxicity, with negative results for mutagenicity and carcinogenicity and only mild eye irritation potential [23,219]. Almost a decade ago, the reported acute, subacute, subchronic, chronic, and carcinogenic studies in rodents exhibited dramatically higher concentrations of SCCPs. In contrast, emerging evidence from contemporary studies indicates that SCCPs at environmentally relevant concentrations can pose significant health risks. While the low acute toxicity is acknowledged, the primary concern has shifted to the effects of chronic, low-level exposure. As summarized in Figure 2, prolonged exposure to SCCPs is associated with adverse outcomes in multiple organ systems, including the liver, kidney, thyroid, and the nervous, endocrine, immune, and hematological systems. Among these, the liver, kidney, and thyroid have been identified as primary target organs. Meanwhile, given that humans are inevitably exposed to SCCPs through diet, inhalation, and dermal contact due to their widespread environmental dispersion [23], a detailed understanding of their organ-specific toxicity is crucial. In this regard, the following sections will therefore systematically summarize and discuss the reported toxic effects of SCCPs on key human organs.

#### 5.1.1. Liver Toxicity

Extensive toxicological research has established the liver as a primary target organ for SCCP toxicity. In vitro studies have shown that SCCPs may activate PPARα-related pathways at relatively high concentrations (≥100 μg/L), suggesting potential mechanisms for hepatotoxicity [156,220,221]. Both subacute and chronic oral exposure in rodents induced dose-dependent increases in liver weight, as well as pathological changes including steatosis, necrosis, inflammation, and vascular alterations.

Studies using human hepatic cell lines (e.g., HepG2) have elucidated potential molecular mechanisms. Exposure to environmentally relevant concentrations of SCCPs (as low as 1–10 μg/L) induced cytotoxicity, showing an increasing trend with increasing carbon-chain length and chlorine constituent, and such results elucidated oxidative stress and remarkable metabolic disruption potentially influencing liver toxicity of SCCPs [14,222]. Recent work has shown that SCCPs can inhibit PPARα-regulated fatty acid oxidation and promote aerobic glycolysis in human hepatocytes at concentrations ≥100 μg/L, although the relevance of PPARα-mediated effects in vitro requires cautious extrapolation to the human liver in vivo [223]. However, hPPARα gene expression in in vitro artificial human hepatocytes, as reported by Gong et al. [223], is much lower than that in human liver, as reported by Kersten et al. [224]. In the same year, Liu et al. confirmed that the concentration of serum SCCPs was positively related to male TB and female aspartate aminotransferase concentrations in residents from Jinan in China, indicating that SCCPs cause hepatotoxicity in human bodies [225]. In 2024, Luo et al. elucidated the effects of SCCPs on human normal hepatic cells, observing remarkable metabolomic perturbations at exposure levels of 1, 10, and 100 μg/L [226]. In human studies, the relationship between SCCP exposure and liver enzyme alterations has been reported in a limited number of studies. However, these findings are observational and should be interpreted cautiously. Overall, current evidence is consistent with potential hepatotoxic effects but is not sufficient to establish causality in humans.

#### 5.1.2. Kidney Toxicity

Kidney toxicity of SCCPs has been well studied in animal studies, particularly through mechanisms involving α2u-globulin accumulation in male rats [220,221,227,228]. Notably, a key study identified a species-specific mechanism where a particular SCCP congener binds to α2u-globulin, a protein abundant in male rat kidneys, triggering its accumulation and consequent nephropathy [227]. However, this mechanism is species-specific and not considered relevant to humans due to the lack of α2u-globulinin in humans. In contrast, recent epidemiological findings from a Chinese cohort reported associations between SCCP exposure and glomerular hyperfiltration. For example, a study of male residents in Jinan, China, found a positive association between serum SCCP concentrations and the risk of glomerular hyperfiltration (GH), an early marker of glomerular injury and potential chronic kidney disease [214]. While these findings are suggestive of potential renal effects, they remain preliminary and require replication and mechanistic clarification.

#### 5.1.3. Thyroid Toxicity

While animal studies suggest that SCCPs may affect thyroid hormone regulation and hematological parameters [13,220,228,229,230], available human studies report the relationships that are subject to potential confusion and methodological limitations. For example, in 2018, Qiao et al. elucidated an obvious increase in SCCP levels in human serum, inducing a significant increase in serum TSH concentrations and leading to a distinguishable reduction trend in T3 content [209]. The finding elucidates that SCCP exposure to the thyroid can reflect the hormonal disruption pattern observed in animal models and indicate a potential risk for thyroid dysfunction in humans.

#### 5.1.4. Developmental Toxicity, Neurotoxicity, and Hematological Toxicity

Beyond organ-specific damage, SCCPs have been linked to broader systemic toxicity, adversely affecting development, the nervous system, and hematological function. While evidence from animal models has highlighted these hazards, recent human studies provide direct evidence of related health risks. Animal studies indicate that SCCP exposure can cause developmental toxicity (e.g., growth inhibition in zebrafish), neurotoxicity (e.g., behavioral alterations and glial cell activation), and hematological disturbances [12,231]. These findings establish a foundation for investigating potential human health impacts. Critically, emerging epidemiological research confirms the relevance of these toxicological endpoints for human populations. A study of residents in Jinan, China, found that measured blood SCCP concentrations were inversely associated with key hematological parameters, including WBC and RBC counts, hemoglobin, hematocrit in males, and hemoglobin indices in females. Such a finding indicates a measurable disruption of hematologic homeostasis associated with SCCP exposure in humans, consistent with concerns raised by experimental toxicology [213]. The results of experimental and human data reveal that SCCP exposure poses risks beyond specific organ damage, potentially affecting critical developmental, neurological, and circulatory functions. Further research is necessary to elucidate the dose–response relationships and mechanisms underlying these systemic effects in exposed human populations.

### 5.2. Toxicokinetics of SCCPs

After oral exposure in the human body, SCCPs are excreted via lung, kidney, and intestine by several typical dynamic procedures, including absorption, distribution, metabolism/biotransformation, and elimination [23]. Animal studies provide insight into the kinetics of these processes. In rats administered a single oral dose, SCCP concentrations in blood peaked approximately 2.8 days post-exposure [232]. Excretion occurs primarily through feces and, to a lesser extent, urine. After 28 days of exposure in rats, about 27.9% and 3.5% of the administered SCCPs were eliminated unmetabolized in feces and urine, respectively, with amounts increasing over time [232]. The physicochemical properties of specific SCCP congeners critically influence their distribution and excretion. The octanol–water partition coefficient (K_ow_) is a key determinant, while the degree of chlorination and carbon-chain length generate the primary excretion route: low-chlorine congeners are more readily excreted in urine, whereas high-chlorine congeners are preferentially eliminated via feces [233,234]. In 2019, Li et al. indicated that SCCPs might experience carbon-chain shortening in plants and confirmed the presence of decomposition isomers containing a CCl_3_ group in pumpkin and soybean seeds [235]. Meanwhile, the degree of chlorination increase could result in enhancement of SCCP aggregation in pumpkin and soybean. Recently, there have been a few studies reporting the toxicokinetics of SCCPs in the human body. For example, Gao et al. utilized an in vitro three-dimensional human skin equivalent (3D-HSE) model to assess relevant percutaneous permeation of SCCPs and confirmed that ~10.2% of SCCPs realized penetration of HSE in 36 h [236]. However, there is still no evidence to verify how SCCPs can permeate the skin barrier through appendageal passage or through the intersecting stratum corneum intercellularly or transcellularly.

To date, the tissue and organ distribution of SCCPs has been intensively investigated in rats, mice, hens, quail, and especially humans using ^14^C-labeled tracers or by analyzing tissue-specific SCCP concentrations [23]. Among the diverse previous studies, the absorbed SCCPs in the human body predominantly exist in fat and organs with strong metabolic activity, such as kidney, liver, brain, hair, and nail, explaining that kidney, liver, brain, and hematopoietic systems are the targets of SCCPs. After the SCCPs are distributed in human tissue and organs via absorption, the metabolism of SCCPs in humans may occur, which can be evidenced by several studies [237,238,239,240]. Since a study reported on C_9_-CPs in maternal and cord serum from women in Beijing, China [209], and investigated the isomers and levels, more enthusiasm has been shown toward the metabolism of SCCPs in humans. In 2020, Dong et al. explored the pharmacokinetic modeling for SCCPs, MCCPs, and LCCPs in rats and humans through in vivo/vitro exposure of S/M/LCCP to rat and liver microsomes, and the relative metabolic rate of SCCPs was estimated to be 1.31 × 10^−6^/h in humans [240]. Meanwhile, the relevant metabolic rate of SCCPs was extremely slow and exhibited a rising trend as the carbon-chain length increased in SCCPs. Whereafter, using multiple computational approaches, Wang et al. investigated SCCP (1-chlorodecane) metabolism by cytochrome P450 enzymes (CYPs) via density functional theory (DFT) calculations, assessed bioaccumulation with regression modeling, and predicted carcinogenicity using the Lazar program [237]. Importantly, corresponding consequences in such a theoretical study indicated that metabolites, 10-chloro-decan-5-ol and 1-chlorodecanol, exhibited much easier bioaccumulation, were more carcinogenic, and caused more cardiovascular injury than parent SCCP. Such work is the first study utilizing theoretical analysis to prove the possible metabolism of representative SCCPs in humans. Soon after, as reported by He et al. and Lin et al. in two in vitro studies [238,239], SCCP concentrations could be confirmed to be reduced drastically after incubation with human liver microsome, indicating that SCCPs could be metabolized by CYP450s, and such results are in contrast to those elucidated by Dong et al. [240]. Comparatively, the number of metabolites in He et al. [238] was higher than that detected in Lin et al. [239], which could be explained by differences in experiment conditions and the extraction pathway. However, it is still not clear which specific isoform or isoforms of CYP450s are involved in SCCP metabolism.

Previous work had indicated that diet was dominating the external exposure of SCCPs to the ordinary populace in north China. Generally, the SCCPs in raw food materials and cooking oil undergo transformation and elimination procedures during thermal procedures of cooking. In this regard, the elimination of SCCPs in food and water appears to be particularly important [162]. In 2018, Ding et al. reported the first theoretical study on the adsorption of representative CPs on the CNT surface in an aqueous environment via DFT and molecular dynamics (MD) methods [241]. Meanwhile, in 2019, Zhang et al. reported the photochemical degradation behavior of 1-chlorodecane (CD, a model of SCCPs) in aqueous solution under UV irradiation [242]. In such work, CD experienced absolute photochemical degradation in 120 min, and •OH could be identified as the primary reactive substance owing to the fact that released Cl• from CD in water could lead to the generation of •OH. In the future, deeper studies should be carried out to realize the elimination of SCCPs in diverse environmental matrices.

### 5.3. Adverse Human Health Effects of SCCPs

In the past, high concentrations of SCCPs, as mentioned in the above sections, could be found in diverse environmental media including wastewater [78], air [132], indoor dust [45], soil [243], and biota [77], owing to the widespread usage and improper disposal of products containing SCCPs. SCCPs could also be identified in human breast milk/blood/placenta [171,190,202,203]. Broadly identified SCCPs in the environment cause possible human health harms via exposure to SCCPs. Accordingly, the assessments of and reduction in SCCP-caused adverse human health effects seem to be important. Risk assessment in previous studies mainly focused on external human exposure to SCCPs through diet, inhalation, and dermal absorption, and discussed the published upper bound of estimated daily intakes (EDIs) near or more than tolerable daily intakes (TDIs) [24]. Moreover, present evidence indicates that non-occupational adult human exposure to SCCPs happens predominantly by diet (~85% relative contribution) and inhalation of indoor air (~15%), while exposure through dust is considered negligible. For young children, however, ingesting dust is considered a more critical exposure approach (~15%), while exposure by diet/inhalation is similar to that of adults. However, the assessments utilizing SCCP concentrations in humans of different ages and genders, including adults (male and female), infants, children, teenagers, and toddlers, are insufficient in identifying the specific health influences of SCCPs.

Current studies elucidate possible adverse health effects of SCCPs in the human body, for example, liver and kidney damage, thyroid hormone, and glucocorticoid imbalances, and so on. Additionally, SCCPs, particularly C_12_-CPs (particularly Cl_6_-SCCPs), have been listed as Group 2B carcinogens by the International Agency for Research on Cancer (IARC). In the following sections, we will summarize the relationship between the exposure to SCCPs and the emergence of detailed diseases in human health problems.

#### 5.3.1. Hepatic Diseases

Past studies have indicated that SCCP exposure to human cells could cause oxidative pressure and therefore modify cellular metabolism and cell viability. At the same time, SCCPs play a role as a peroxisome proliferator and obstruct lipid metabolism. Geng et al. evaluated the relevant influence of SCCP dose on metabolic response of human hepatoma HepG2 cells, which serve as metabolically related models for in vitro hepatotoxicity research, and investigated oxidative stress induction, metabolic alteration, and related metabolic enzyme activities [222]. In this work, HepG2 cells were firstly incubated with various levels of C_10_-SCCPs (1, 10, 100 μg/L; Cl, 60.9%) for 24 and 48 h, respectively, and the results presented a decrease in cell viability, variation in the intracellular redox state, and disorder in metabolite curves, suggesting that human hepatoma may be caused via environmental dose-related SCCP exposure ranging from 1 to 100 μg/L. In 2024, Luo et al. investigated metabolomic interference in human normal hepatic (L02) cells after exposure to SCCPs with doses of 1 μg/L, 10 μg/L, and 100 μg/L via employing metabolomics technology [226].

#### 5.3.2. Thyroid Diseases

According to previous studies, corresponding concentrations of five THs, such as T3, T4, free triiodothyronine (FT3), free thyroxine (FT4), and thyroid-stimulating hormone (TSH), can influence relevant functioning of nearly all tissues via relevant effects on cell metabolism, and higher THs present the possibility to induce the sympathetic nerve, stimulation, and hypermetabolism, generating palpitations, sweating, and weight loss [244,245]. Meanwhile, toxicology studies elucidated that CPs could disturb TH homeostasis and initially established the associations between SCCPs and TH levels. For example, Qiao et al. researched mass fractions and congener group patterns of SCCPs/MCCPs in paired maternal and cord serum to explore the placental transport mechanism and prenatal exposure risks of SCCPs [209]. In such work, relationships between SCCPs and TH contents in maternal and cord serum were estimated through correlation analysis, and relative consequences exhibited a remarkable positive correlation between ΣSCCP mass fractions and TSH contents in maternal serum, which indicated that exposure to SCCPs could influence rotative TH concentrations in humans and thus have some influence on human health.

#### 5.3.3. Diabetes

The primary characteristic of diabetes is hyperglycemia, originating from insulin resistance or pancreatic beta cell dysfunction [246], and diabetes has been considered a global public health priority because of rapid growth, prevalence, and disease burden. As a representative of diabetes, type 2 diabetes (T2Ds) is not only a crucial risk factor for increasing global mortality worldwide but is also a nonnegligible incentive of other diseases. Recently, Zhang et al. reported that human exposure to SCCPs could boost the relevant risk of T2Ds and destroy lipid metabolism [247]. In this work, 344 participants (172 diabetics and 172 healthy controls individually matched on sex and age) aged 25–80 years with no family history of diabetes were recruited. The overall joint effects of SCCP mixture exposure on type 2 diabetes risk and lipid parameters were estimated after adjusting for age, sex, BMI, alcohol consumption, and smoking status. Corresponding results of such a study showed that C_10–11_-CPs and ΣSCCPs had a positive correlation with the relevant risk of T2Ds, representing a nonlinear dose–response correlation. Long-term exposure to SCCPs could lead to a higher risk of T2Ds in males and obese people. Exposure to C_10_-CPs and C_11_-CPs was positively correlated with lipid parameters, indicating that SCCPs could facilitate relevant evolution of T2Ds through destroying lipid homeostasis. Meanwhile, SCCP exposure to pregnant women can cause gestational diabetes mellitus (GDM), and GDM can be considered a remarkable type of diabetes that develops during pregnancy, which has been elucidated by Yang et al. [21]. In this study, the authors performed a promising investigation utilizing a nested case–control design to estimate the potential association between SCCP exposure during pregnancy and risk of GDM. To identify relevant associations, serum samples from 102 pregnant women diagnosed with GDM and 204 healthy controls were gathered in Hangzhou, Eastern China. The criteria for selecting the eligible participants were as follows: (1) no history of diabetes or family history of diabetes; (2) no history of liver disease, genetic disease, or cancer; (3) single birth; and (4) available information on epidemiology. It can be found that the median concentration of SCCPs in the GDM family (161 ng/mL) is distinctly larger than that in the non-GDM family (127 ng/mL). Meanwhile, two specific SCCP congeners, C_13_H_23_Cl_5_ and C_10_H_16_Cl_6_, were identified as key variants, showing a significant positive association with GDM risk. The results further indicated that SCCP congeners with lower-chlorine content induced more pronounced glycemic disruption and higher GDM risk. This might be because lower-chlorine SCCPs, with their reduced spatial resistance and molecular weight, more readily cross cell membranes and interact with biomolecules or target proteins to exert biological effects. Recent studies in China have reported a relationship between SCCP exposure and metabolic outcomes, including diabetes and gestational diabetes. However, these findings are based on specific study designs (e.g., cross-sectional or nested case–control), with limited sample sizes. Therefore, potential influence factors, including co-exposure to other pollutants and lifestyle variables, should be carefully considered when interpreting these results.

As discussed in the above-mentioned content, our review concluded a novel perspective about the risk of SCCP exposure faced by the Chinese population and disclosed the potential seriousness of SCCP distribution in China. Moreover, our review and past works identified that professional workers and indigenous inhabitants possessed a high risk of exposure to SCCPs originating from industrial activities [38]. Meanwhile, a recent study evaluates the association between PM_2.5_-bound SCCPs and asthma, along with relative symptoms, in school-aged children and adolescents, confirming that C_11_-, C_12_-SCCPs contributed the most positive weight to the risk of asthma and relative symptoms [248]. However, human evidence remains limited and largely depends on observational studies reporting correlation rather than causal relationships. Further longitudinal and mechanistic studies are required to establish causal links and better understand the health implications of SCCP exposure.

## 6. Control and Treatment Technologies for SCCPs

Given the high environmental levels summarized in Section 2, this section reviews current and emerging strategies for mitigating SCCP contamination. We cover source control and regulatory alternatives (Section 6.1), including green chemical substitution (Section 6.1.1) and China’s evolving regulatory framework (Section 6.1.2). We then compare physicochemical treatment methods (adsorption, reductive dechlorination, photocatalysis) and bioremediation approaches (microbial degradation, phytoremediation, combined remediation), presenting their relative advantages and limitations in Table 11.

### 6.1. Source Control and Alternative Strategies

Numerous studies have indicated that exposure to SCCPs could cause serious adverse health effects, including but not limited to liver and kidney damage, endocrine disorders, and reproductive and developmental toxicity. Considering these effects, regulatory action has been taken on national and global scales to evaluate and manage the environmental and health risks posed by SCCPs. Notably, SCCPs and MCCPs have been listed and recommended to be listed as POPs under the Stockholm Convention since 2017 and 2024, respectively [249,250]. Due to their diverse structures and wide varieties, SCCPs exhibit complex behavior and characteristics in the environment. In addition, given their hidden environmental presence, the traditional remediation techniques have been elucidated to be less effective in treating these SCCPs. Accordingly, strengthening control measures from the source and actively promoting the development and application of green alternatives are crucial for effectively preventing and mitigating the environmental and health risks posed by SCCPs. The essential approach to substitution strategy relies on the molecular structure optimization to remarkably decrease the persistence, bioaccumulation, and toxicity (PBT) characteristics of chemicals while keeping their outstanding inherent properties, thereby achieving a synergistic optimization of the environmental friendliness and economic benefits of chemicals.

#### 6.1.1. Green Chemical Substitution

Following the global ban on SCCPs under the Stockholm Convention in 2017 and 2024, alternatives like MCCPs, LCCPs, thiophosphates, and bio-based materials have been adopted. Although MCCPs serve as effective substitutes, their use releases SCCP impurities into the environment, and they present a higher bioaccumulation potential than SCCPs, despite lower inherent toxicity [251]. Among current alternatives, MCCPs have been listed as a Substance of Very High Concern (SVHC) by the EU and are proposed for inclusion in the Stockholm Convention. Other substitutes also face challenges: thiophosphate esters like triphenyl thiophosphate (TPPT) offer superior lubricant performance but degrade into ecotoxic byproducts [252]; chlorinated bio-based plasticizers such as epoxidized soybean oil (ESO) are more environmentally friendly substitutes for CPs yet generate contaminated wastewater, limiting their use. This dilemma between functionality and environmental safety indicates the urgent need for high-performance, sustainable alternatives to SCCPs.

Strict global regulations and market pressures are prompting the urgent need for environmentally benign chemical alternatives, a dual imperative for industrial modernization and ecological sustainability. This calls for a comprehensive evaluation framework that simultaneously assesses environmental safety, technical performance, and economic viability. To succeed, cross-disciplinary innovation and robust global governance must work in concert to avert the adoption of “regrettable substitutes” that solve one problem while creating new systemic risks.

#### 6.1.2. The Regulatory Framework for Industrial Emissions

In an effort to protect human health and the environment, major economies, including the EU, the U.S., Japan, and China, have implemented regulations governing new pollutants. These rules aim to control their emissions and residues across industrial and consumer sectors. Given the presence of SCCPs in diverse products, regulatory approaches vary globally. The EU (Regulation (EU) 2019/1021) and Switzerland (Chemical Risk Reduction Ordinance) enforce strict prohibitions, banning the manufacture and sale of substances/mixtures containing ≥1% SCCPs (CAS 85535-84-8) and articles containing ≥0.15% SCCPs. China has listed SCCPs in its “List of Key-Controlled New Pollutants (2023 Edition)” for enhanced management, effective 1 March 2023. In contrast, the United States and Japan have not yet established an individual national standard with specific numerical limits for SCCPs in CP products.

In the textile industry, ISO 22818:2021(en) provides a key analytical method (GC-NCI-MS) for determining SCCPs and MCCPs, which are used as flame retardants, plasticizers, and finishing agents. While this standard does not set product limits, specific regulations also do. For instance, the EU regulations on POPs enforce a 0.15% limit on SCCPs in textile components. Similarly, China has proposed amending its mandatory standard GB 21027-2020 to limit SCCPs in contactable plastic parts of student stationery to <1.5 g/kg, with testing to follow GB/T 33345.

### 6.2. Environmental Treatment Technologies

#### 6.2.1. Physicochemical Methods

In recent years, various methods for SCCP treatment have been reported, primarily including physicochemical and biological approaches. Physicochemical methods play a key role in the remediation of SCCPs via utilizing both physical and chemical procedures to degrade, remove, or immobilize SCCPs. Corresponding removal mechanisms mainly involve adsorption, redox reactions, chemical degradation, and photodegradation. These technologies are considered effective for the removal or mineralization of SCCPs.

(1) Conventional physicochemical treatment

According to the environmental persistence of SCCPs, adsorption utilizing appropriate adsorbents, such as activated carbon, biochar, metal–organic frameworks (MOFs), and functionalized nanomaterials, represents a theoretically promising removal strategy. However, no relative studies on this approach have been reported to date. In addition, membrane separation technology, based on selective permeability, may be a feasible strategy for removing SCCPs. For low-energy removal of particles and colloids, ultrafiltration (0.01–0.1 μm) is effective, though limited for small molecules. Nanofiltration (1–2 nm) enhances organic pollutant removal but requires attention to fouling and cost. Reverse osmosis (<1 nm) achieves the highest effluent quality, making it suitable for heavily polluted water, yet its significant energy needs and brine management complexity constrain large-scale adoption [253].

Under suitable conditions, SCCPs in the environment can realize dechlorination. Lahaniatis et al. [254] found that sodium could induce the reductive dechlorination of CPs in an ammoniated diethyl ether solution, with the identified degradation products being n-alkanes and n-alkenes. In a study on the reductive dechlorination and removal of SCCPs using nano-zero-valent iron (n-Fe^0^) particles, Zhang et al. [255] observed that the dechlorination rate was regulated by pH, n-Fe^0^ dosage, temperature, and the concentration of humic acid. The addition of an appropriate amount of humic acid promoted the reductive dechlorination of SCCPs by n-Fe^0^. However, if the relative concentration of n-Fe^0^ was higher than 15 mg/L, the dechlorination rate was inhibited. Although physicochemical methods such as n-Fe^0^ treatment achieve high SCCP removal efficiency, their high cost and operational complexity necessitate further improvement.

(2) Catalytic degradation

Compared to general physical treatment strategies, catalytic degradation, particularly thermal and photocatalytic procedures, presents desirable cost-effectiveness, higher efficiency, and less secondary pollution, verifying the prospective potential of SCCPs.

**Figure 3:**
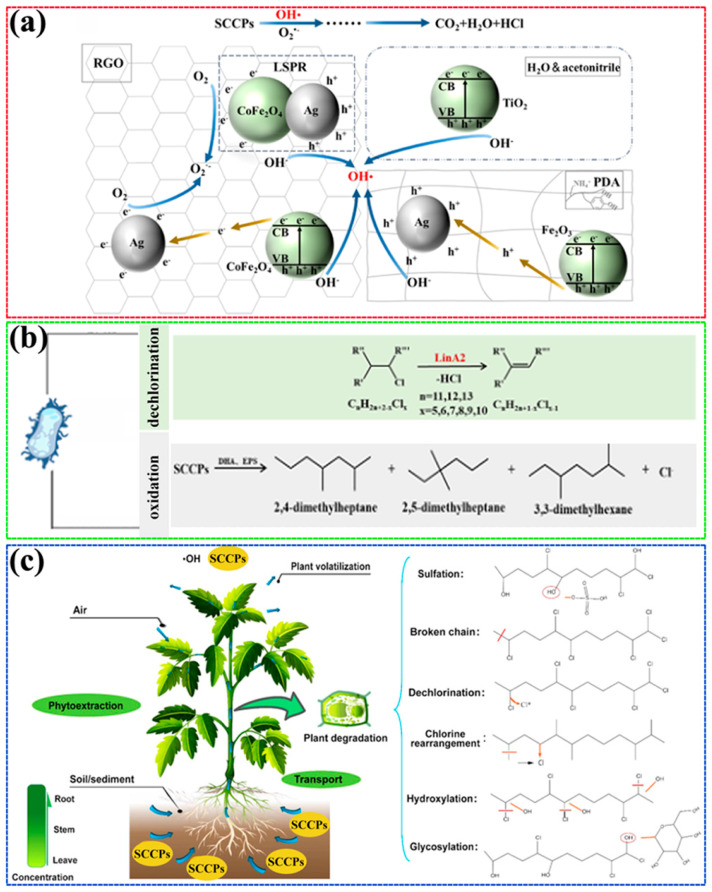
(**a**) Photocatalysis degradation mechanisms of SCCPs [266]; (**b**) microbial degradation mechanisms of SCCPs [266]; (**c**) the main mechanisms and reaction processes of SCCP degradation by plants [274].

Due to the lack of appropriate substituent groups to absorb ultraviolet light >290 nm, SCCPs generally do not generate direct photocatalytic activity under environmental conditions. SCCPs exhibit excellent thermal and chemical stability, with minimal hydrolysis and oxidation occurring in aqueous phases at room temperature. However, in aquatic environments where radicals or catalysts exist, hydrolysis or oxidative photocatalytic reactions can be induced. SCCPs may also undergo indirect photodegradation by being attacked by oxidative radicals in the troposphere. In recent years, advanced oxidation technologies such as photocatalysis (semiconductors like TiO_2_ [256], ZnO [257], Cu_2_O [258], silver salt composites [259], bismuth composites [260], non-metal semiconductors [261]) and Fenton-based [262,263] processes have rapidly developed for the removal of POPs from the environment. The semiconductors employed for photocatalysis present a unique band structure that forms photogenerated electrons and holes under light absorption, prompting redox reactions to degrade pollutants. These technologies can either directly mineralize pollutants or enhance their biodegradability through oxidation. Currently, studies on photocatalytic degradation materials for SCCPs remain limited, with reported studies primarily concerning two composite materials: reduced graphene oxide (RGO)/CoFe_2_O_4_/Ag and Fe_2_O_3_@polydopamine (PDA)-Ag (Figure 3a) [264,265,266]. The remarkable performance of these photocatalytic materials originates from their ability to promote charge carrier separation, thereby generating reactive O species that mineralize SCCPs. The general mechanism involves photogenerated electrons reducing O_2_ to ·O_2_^−^ and holes oxidizing H_2_O to ·OH, which synergistically degrade SCCPs to CO_2_, H_2_O, and HCl. This principle is exemplified by RGO/CoFe_2_O_4_/Ag, which achieves a 91.9% degradation rate in 12 h (× higher than TiO_2_ P25) due to Ag’s plasmonic effect and graphene’s conductivity, enhancing e^−^/h^+^ separation [264]. Likewise, in Fe_2_O_3_@PDA-Ag hybrids, Ag nanoparticles accumulate electrons and the PDA shell improves charge transfer, resulting in efficiency improvements of 1.3~2.9 times over control materials. Furthermore, DFT calculations provide molecular-level insight, revealing that mineralization proceeds via ·OH-mediated H abstraction followed by sequential dichlorination (Equations (1) and (2)).

SCCP + ·OH→ SCCP + H_2_O (1)

·SCCP + ·OH→ HCl + H_2_O + CO_2_ (2)

Although photocatalysis processes can be applied to degrade SCCPs, their efficiency is often constrained by initial contaminant concentration and reaction conditions. Moreover, photocorrosion represents a typical drawback of photocatalysis, as semiconductor photocatalysts are generally unstable under light irradiation. Therefore, exploring more efficient and environmentally friendly methods for mineralizing SCCPs, as well as seeking other cost-effective degradation technologies, is of critical importance.

#### 6.2.2. Bioremediation Technologies

Unless otherwise specified, the bioremediation technologies discussed below are drawn from studies on SCCPs themselves. Where evidence for SCCPs is unavailable, we draw on studies of structurally or behaviorally similar contaminants (e.g., other POPs such as atrazine and BDE-209); such cases are explicitly noted, and the potential transferability of findings is discussed. Compared to physicochemical methods, bioremediation, as the most economical approach, utilizes the metabolic functions of organisms to reduce the concentration of pollutants in the environment or make them harmless through biosorption or biodegradation and is considered one of the most reliable strategies for reducing organic contaminants from the environment, presenting remarkable advantages in environmental friendliness, cost-effectiveness, and sustainability [267]. Moreover, bioremediation is usually categorized into microbial remediation, phytoremediation, and combined remediation, based on the involved organisms [268].

(1) Microbial remediation

Some microorganisms can remove hazardous pollutants from the environment through biodegradation, with their intrinsic microbial metabolism serving as the primary biodegradation mechanism [269,270]. Studies have confirmed that halogenated compounds can act as carbon and energy sources for specific aerobic microorganisms. Therefore, obtaining microorganisms with degradation capabilities is a prerequisite for microbial remediation approaches.

Microbial removal is a strategy that involves screening and acclimating specific microorganisms to degrade organic pollutants in the environment, while microbial degradation itself is also one of the essential processes for breaking down organic pollutants via the pathways of dechlorination and oxidation, as described in Figure 3b [266]. It is well known that CPs are compounds resistant to degradation by common microorganisms but can be broken down and removed by specific microbes. The degradation process is highly influenced by temperature, pH, chain length, chlorination degree, stereoisomers, microbial strains, and environmental conditions, and SCCPs with a Cl degree below 60% are particularly susceptible to microbial oxidation [271]. In recent years, microbial degradation has shown promising potential in the treatment of SCCPs. Common microorganisms utilized for the degradation of contaminated regions include bacteria, fungi, microalgae, or microbial consortia [269,270]. Moreover, the addition of nutrients or specific chemical reagents to the bio-culture procedure can accelerate the degradation of SCCPs. Specifically, adding low concentrations (20 mg L^−1^) of glucose, Tween-80, or acetone to the culture medium has been shown to be effective. Here, Tween-80 acts as a surfactant, improving the solubility and dispersion of SCCPs and thereby increasing their bioavailability to microbes. Furthermore, maintaining a moderate, near-neutral pH creates the most favorable conditions for this microbial degradation [272].

(2) Plant absorption

Plants can absorb pollutants from soil or water through passive or active uptake by their roots, where processes such as accumulation, degradation, mineralization, and phytovolatilization can occur [273,274], as summarized in Figure 3c. For instance, non-ionic pollutants primarily enter plants via passive uptake, driven by the electrochemical potential gradient between the interior and exterior of the roots. Additionally, plants can absorb atmospheric pollutants through their leaves. Li et al. [235,275,276] selected soybean and pumpkin as model plants and, through indoor hydroponic exposure experiments, discovered a carbon-chain cleavage process of SCCPs mediated by plants, which was an SCCP-specific example. Under the influence of soybean and pumpkin seedlings, highly chlorinated SCCP congeners faced dechlorination to form lower-chlorinated congeners, accompanied by molecular rearrangement of chlorine atoms. In the tissues of both plants, dechlorination and chlorine rearrangement products, such as the daughter chlorodecanes C_10_H_17_Cl_5_, C_10_H_16_Cl_6_, and C_10_H_15_Cl_7_, were detected, along with carbon cleavage products. In comparison, soybean exhibited faster translocation and degradation of the parent SCCPs than pumpkin, as well as a greater extent of degradation, whereas pumpkin accumulated higher levels of the parent SCCPs than soybean. Although real soil, plant systems differ from hydroponic exposure systems, where SCCPs are often adsorbed onto soil and may have lower bioavailability. These findings provide important insights into the phytodegradation of SCCPs in the environment.

(3) Combined remediation

Combined remediation integrates multiple technologies to address the shortcomings of single-method approaches. Plant–microbe remediation is particularly prominent, capitalizing on a synergistic relationship where microbial metabolites and root exudates jointly enhance contaminant bioavailability and mutual growth. This synergy is exemplified and strengthened in mycorrhizal symbiotic systems. Here, bidirectional nutrient exchange pathways significantly boost plant stress resistance and microbial activity, creating a highly effective bioremediation platform. Direct evidence for combined remediation of SCCPs is currently lacking. However, insights can be drawn from studies on structurally similar persistent organic pollutants. For example, studies on atrazine showed that the arbuscular mycorrhizal fungus Glomus caledonium promoted its accumulation and metabolism in maize and enhanced degradation by altering soil properties [277,278]. Similarly, for BDE-209 (a brominated flame retardant with physicochemical properties comparable to SCCPs), mycorrhizal inoculation improved degradation in ryegrass [279]. These findings suggest potential transferability to SCCPs, as mycorrhizal fungi can enhance plant uptake of hydrophobic organic contaminants through improved root surface area, altered root exudation, and stimulation of microbial degradation in the rhizosphere. However, direct experimental confirmation using SCCPs as target contaminants is urgently needed before recommending combined remediation as a practical strategy for SCCP-contaminated sites. Further supporting this potential, inoculation with Glomus etunicatum increased aromatic carbon content in alfalfa roots, leading to greater phenanthrene absorption [280]. The potential of combining mycorrhizae with surfactants has also been demonstrated, as surfactants improve contaminant desorption and contact with biological agents [281].

#### 6.2.3. Comparison of the Advantages and Disadvantages of Each Remediation Method

The remediation effectiveness of SCCPs varies across different methods. Table 11 compares the advantages and disadvantages of various approaches, as well as the influencing factors and possible mechanisms involved in SCCP removal. As shown in Table 11, pH and temperature are key factors affecting SCCP removal regardless of the strategy applied. Physicochemical methods generally achieve high removal efficiency for SCCPs; however, they often require complex and stringent operating conditions and higher costs. In contrast, the effectiveness of biological methods largely depends on the degradation and adsorption capabilities of the organisms used. Overall, biological treatments typically involve longer processing cycles and are more susceptible to external environmental interferences. Notably, while combined plant–microbe remediation has shown promise for structurally related POPs (e.g., atrazine, BDE-209), direct evidence for SCCP remediation using these approaches remains limited. Transferability is plausible due to shared hydrophobicity and persistence, but SCCP-specific validation studies are a critical research gap (see Section 7).

**Table 11:** Comparison of the environmental remediation technologies of SCCPs.

Technologies	Effect Factors	Advantages	Disadvantages	Reaction Pathways/Mechanisms	Reaction Products
Conventional physicochemical treatment [242,255]	Dosage, concentration, pH, temperature, carbon-chain length, and chlorination degree of SCCPs and catalysts	A simple process with a high pollutant removal rate	High treatment costs and unsuitable for large-scale application	Reductive dechlorination	Normal alkanes and normal alkenes, alcohols, or long-chain intermediates
Catalytic degradation [264,265]	Light intensity, dosage, concentration, pH, and temperature of SCCPs and catalysts	Short time consumption, high efficiency, good reproducibility, easy to handle, and convenient for engineering applications	High cost, difficult to regenerate photocatalysts, complex processing components, and prone to generating organic byproducts	Photocatalytic degradation	Intermediates of olefins and carbonyl compounds, H_2_O, CO_2,_ and HCl
Microbial remediation [272]	Carbon-chain length, degree of chlorination, pH, and temperature	Low cost, simple operation, and no secondary pollution	Long degradation cycle of bacteria	Biotransformation and dechlorination degradation	Low-chlorine analogs or normal alkanes
Plant absorption [275]	Carbon-chain length, degree of chlorination, pH, and temperature	Low cost and environmentally friendly	Plant cultivation is relatively slow and not suitable for large-scale application	Dehalogenation and hydroxylation, dechlorination, and chlorine rearrangement in plant tissues	Low-chlorine analogs, such as C_10_H_17_C_l5_, C_10_H_16_C_l6,_ and other C_10_H_15_C_l7_
Plant–microbe combined remediation [277]	Carbon-chain length, degree of chlorination, pH, and temperature	Enhance contaminant bioavailability, mutually stimulate growth and activity, and boost plant stress resistance and microbial activity	System complexity, site-specificity, and time-consuming	Combination of microbial remediation and plant absorption	Low-chlorine analogs or normal alkanes

## 7. Summary and Future Outlooks

This concluding section clarifies the main findings of the review and identifies priority research and policy needs. As we all know, China started producing CPs in the mid-20th century, and CPs can be ubiquitously identified in multifarious environmental matrices, animals, human foods, and human bodies. Even if being viewed as POPs, the relevant production and/or consumption of SCCPs in China still continues. In this regard, the Chinese government should participate in the reduction in and even elimination of SCCPs from diverse environmental matrices. In this review, our primary concern is the recent research progress in the distribution levels, detection, toxicity, and adverse human health effects of SCCPs in China published between 2015 and 2025. Specifically, most studies focused on SCCPs presenting the main contents, including (1) distribution and levels of SCCPs in environment matrices of China, for example, air, water, soil, sediments, biota, and food; (2) development of SCCP determination methods, which help quantify SCCPs correctly; (3) relevant distribution of SCCPs in human bodies, such as human placenta, breast milk, blood, and organs (fat, kidney, liver, brain, bone, etc); (4) toxicity, toxicokinetics, and adverse health effects of SCCPs in humans; (5) current development of control and treatment technologies for SCCPs. However, there are several critical issues particularly related to China that need to be considered in future studies.

(1) Population-based studies on SCCPs in China may be limited by selection bias and population heterogeneity, such as children, occupational workers, and pregnant women, which could lead to the misinterpretation of health effects. This necessitates large-scale efforts to quantify body burdens and establish correlations with clinical outcomes.
(2) Because of improper location selectivity during industrial construction, especially the e-waste, textiles, and plastic industries, commercial CPs normally contain indistinguishable mixtures of S/M/LCCPs. Confirming corresponding differences in the toxicity of single CP congeners and building models or calculation methodologies to assess total toxic equivalents would be particularly imperative.
(3) The complexities of SCCP analysis, particularly due to the isomeric diversity and the difference in characterization tools, standards, and data processing strategies, require a comparable and accurate quantification, which is crucial for combining knowledge of different laboratories dealing with potential environmental risks from SCCPs.
(4) Further laboratory studies are indispensable, especially studies using human-related concentration levels and long-term exposure. Novel technical strategies containing omics, in vitro models, and in silico simulations could be extremely helpful in disclosing possible adverse health effects of SCCPs. In addition, the joint effect of SCCPs with other pollutants, for example, polycyclic aromatic hydrocarbons (PAHs), requires more attention.
(5) Compared to other countries in the world, it can be found that China represents the most severe exposure to SCCPs in all dietary food types, even in non-industrial regions. To support China’s New Action Plan and Stockholm Convention commitments, future studies should prioritize the following: (a) establishing baseline SCCP concentrations in all major environmental and human matrices nationwide; (b) developing standardized analytical protocols to enable inter-laboratory comparability; (c) conducting longitudinal cohort studies to clarify health effects, especially in vulnerable groups; (d) quantifying emission sources and pathways to inform targeted controls; and (e) accelerating the development and validation of green substitutes with lower persistence, bioaccumulation, and toxicity.
(6) The priority targets of SCCPs are still unknown, which is a key bottleneck in current toxicology studies. Although state-of-the-art omics technology, including transcriptomics and metabolomics, can identify hundreds to thousands of genes, proteins, or metabolites synchronously, it is still a long way from effectively confirming relevant interactions between chemicals and toxic targets.
(7) Long-term exposure assessment: lack of cohort-based epidemiological studies.
(8) Artificial intelligence (AI) presents significant promise in SCCPs. It can enhance detection by rapidly analyzing complex chromatographic data for accurate identification. AI-driven models, such as QSAR and molecular docking, help predict the toxicity and environmental behavior of different SCCP congeners. Machine learning optimizes degradation strategies by screening effective catalysts or microbial strains. Additionally, generative AI aids in designing greener alternatives with lower environmental impacts. These AI applications support smarter monitoring, risk assessment, and sustainable management of SCCPs.

## Figures and Tables

**Table 1:** SCCP concentrations in air, water, soil, sediment, biota, and food samples collected from various regions across China (note: dw = dry weight; ww = wet weight; ND = not detected; NA = not available).

Region	Air (ng/m^3^)		Water (ng/L)	Soil (ng/g dw)	Sediment (ng/g dw)	Biota (ng/g dw)	Food (ng/g ww)
	Gas Phase	Particle Phase					
Eastern China	6.08–333	9.8–1442	7.4–1978	ND-554,161	ND-16,000	ND-30,000	ND-649
Southern China	1.11–39.8	0.832–109	61.0–460	ND-5090	ND-350,000	11.1–70,400	17–69
Northern China	9.77–1350	16.9–87.7	20–56,306	121–5159	131.8–8700	320–4270	11.7–248
Central China	NA	NA	1131–65,640	NA	4.19–397,600	NA	NA
Southwestern China	1.01–14.4	NA	NA	0.22–948	NA	NA	ND-42,900
Northeastern China	4.04–78	15.1–168	4.1–1490	24.8–1824	64.9–1683	374–1700	NA
China Sea areas	NA	3.31–30.4	12.2–430	NA	9.38–1757	9.3–9100	NA
Background area	0.13–1.27	NA	NA	56.8–1348	NA	3.9–107	NA

## Data Availability

No new data were created or analyzed in this study. Data sharing is not applicable to this article.
